# Single cell RNA-seq reveals cellular and transcriptional heterogeneity in the splenic CD11b^+^Ly6C^high^ monocyte population expanded in sepsis-surviving mice

**DOI:** 10.1186/s10020-024-00970-0

**Published:** 2024-11-06

**Authors:** Haruki Watanabe, Minakshi Rana, Myoungsun Son, Pui Yan Chiu, Yurong Fei-Bloom, Kwangmin Choi, Betty Diamond, Barbara Sherry

**Affiliations:** 1 Institute of Molecular Medicine, Feinstein Institutes for Medical Research, Northwell Health, 350 Community Dr., Manhasset, NY 11030 USA; 2 Department of Molecular Medicine, Donald and Barbara Zucker School of Medicine at Hofstra/Northwell, Hempstead, NY 11549 USA; 3 Division of Experimental Hematology and Cancer Biology, Cincinnati Children’s Hospital Medical Center, 3333 Burnet Ave, Cincinnati, OH 45229 USA; 4 Arthritis and Tissue Degeneration Program, Hospital for Special Surgery at Weill Cornell Medicine, New York, New York 10021 USA

**Keywords:** Sepsis, CLP, CD11b^+^Ly6C^high^, Phagocytosis, Glycolysis, scRNA-seq

## Abstract

**Background:**

Sepsis survivors exhibit immune dysregulation that contributes to poor long-term outcomes. Phenotypic and functional alterations within the myeloid compartment are believed to be a contributing factor. Here we dissect the cellular and transcriptional heterogeneity of splenic CD11b^+^Ly6C^high^ myeloid cells that are expanded in mice that survive the cecal ligation and puncture (CLP) murine model of polymicrobial sepsis to better understand the basis of immune dysregulation in sepsis survivors.

**Methods:**

Sham or CLP surgeries were performed on C57BL/6J and BALB/c mice. Four weeks later splenic CD11b^+^Ly6C^high^ cells from both groups were isolated for phenotypic (flow cytometry) and functional (phagocytosis and glycolysis) characterization and RNA was obtained for single-cell RNA-seq (scRNA-seq) and subsequent analysis.

**Results:**

CD11b^+^Ly6C^high^ cells from sham and CLP surviving mice exhibit phenotypic and functional differences that relate to immune function, some of which are observed in both C57BL/6J and BALB/c strains and others that are not. To dissect disease-specific and strain-specific distinctions within the myeloid compartment, scRNA-seq analysis was performed on CD11b^+^Ly6C^high^ cells from C57BL/6J and BALB/c sham and CLP mice. Uniform Manifold Approximation and Projection from both strains identified 13 distinct clusters of sorted CD11b^+^Ly6C^high^ cells demonstrating significant transcriptional heterogeneity and expressing gene signatures corresponding to classical-monocytes, non-classical monocytes, M1- or M2-like macrophages, dendritic-like cells, monocyte-derived dendritic-like cells, and proliferating monocytic myeloid-derived suppressor cells (M-MDSCs). Frequency plots showed that the percentages of proliferating M-MDSCs (clusters 8, 11 and 12) were increased in CLP mice compared to sham mice in both strains. Pathway and UCell score analysis in CLP mice revealed that cell cycle and glycolytic pathways were upregulated in proliferating M-MDSCs in both strains. Notably, granule protease genes were upregulated in M-MDSCs from CLP mice. ScRNA-seq analyses also showed that phagocytic pathways were upregulated in multiple clusters including the classical monocyte cluster, confirming the increased phagocytic capacity in CD11b^+^Ly6C^high^ cells from CLP mice observed in ex vivo functional assays in C57BL/6J mice.

**Conclusion:**

The splenic CD11b^+^Ly6C^high^ myeloid populations expanded in survivors of CLP sepsis correspond to proliferating cells that have an increased metabolic demand and gene signatures consistent with M-MDSCs, a population known to have immunosuppressive capacity.

**Supplementary Information:**

The online version contains supplementary material available at 10.1186/s10020-024-00970-0.

## Introduction

Sepsis is defined as life-threatening organ dysfunction caused by a dysregulated host response to infection (Singer et al. 2016). In the US at least 1.7 million adults develop sepsis, and 270,000 die from the disease, each year (https://www.cdc.gov/sepsis/what-is-sepsis.html). Two features of the dysregulated host response to sepsis are suppression of innate immunity and alterations in the number, localization, and function of a variety of immune cells, both of which persist for years in those who survive a septic episode and contribute to poor long-term outcomes.

The identification of the mechanisms that contribute to sustained immune dysfunction in sepsis survivors is an area of active study. One contributing factor is believed to be profound alterations in monocyte/macrophage effector cell function that result in failure to respond appropriately to pathogen challenge (Delano and Ward 2016a, 2016b). Monocytes are innate immune cells with high plasticity and heterogeneity. In both mice and humans, postnatal monocytes arise from the common myeloid progenitors, which also give rise to neutrophils, and conventional and plasmacytoid dendritic cells (DCs) (Yanez et al. 2017; Hettinger et al. 2013; Akashi et al. 2000; Liu et al. 2019; Fogg et al. 2006). Under homeostatic conditions monocytes are released into the bloodstream, move into tissues, and differentiate to replenish macrophages, and play an integral role in shaping inflammation and its resolution in tissues (Ginhoux and Jung 2014; Tacke et al. 2007; Geissmann et al. 2010; Menezes et al. 2016). Monocytes play key roles in the host response to pathogens through phagocytosis and cytokine production, orchestrating innate and adaptive immunity (Geissmann et al. 2010). Monocyte-derived dendritic cells (MoDCs) differentiate from monocytes in response to inflammation and infection (Schlitzer et al. 2015).

In the steady state, the bone marrow and other extramedullary locations, like the spleen and lungs, contain monocyte reserves that can be mobilized to other tissues (Swirski et al. 2009). Extramedullary hematopoiesis in response to inflammatory and viral stimuli has been observed in the adult mouse spleen (Loukov et al. 2016). In humans, extramedullary hematopoiesis is uncommon and occurs mainly in patients with myeloproliferative neoplasms (Cenariu et al. 2021). Myeloid-derived suppressor cells (MDSCs) are a heterogenous population of immature myeloid cells (IMCs) with immunosuppressive properties that expands as result of inflammation (“emergency myelopoiesis”) in sepsis (Cuenca et al. 2011). There have been reports of expansion of MDSCs and alteration in other cell types, including DCs, in both patients with sepsis and in animal models of sepsis. (Brudecki et al. 2012; Schrijver et al. 2019; Zhang et al. 2023). There are two types of MDSCs, polymorphonuclear MDSCs (P-MDSCs) and monocytic MDSCs (M-MDSCs), that are morphologically and phenotypically distinct. Both types express the myeloid lineage marker CD11b, but M-MDSCs are Ly6C^high^ Ly6G^−^, while P-MDSCs are Ly6C^low/−^Ly6G^+^ (Bronte et al. 2016).

In mice, there are two major subsets of circulating monocytes. The first subset comprises “inflammatory” or classical monocytes that are CD11b^+^ Ly6C^high^ and express the CC chemokine receptor 2 (CCR2) at an elevated level. This subset corresponds to CD14^+^CD16^−^ monocytes in humans. The second subset comprises “resident” or patrolling monocytes that are CD11b^+^ Ly6C^low^ and express a low level of CCR2, but an elevated level of C-X3-C motif chemokine receptor 1 (CX3CR1). This subset corresponds to CD14^lo^CD16^+^ monocytes in humans (Geissmann et al. 2003; Tsou et al. 2007). In sepsis, multiple studies have shown monocyte heterogeneity and expansion with immune alterations, including enhanced phagocytosis (Zhang et al. 2024, 1994; Yao et al. 2023; Liddiard et al. 2011; Wong et al. 2012; Swan et al. 2007). We and others have reported a marked expansion of splenic CD11b^+^Ly6C^high^ monocytes in the CLP murine model of polymicrobial sepsis that is sustained in animals that survive (Valdes-Ferrer et al. 2013; Rana et al. 2018).

Phagocytosis is the process of ingestion of particles larger than 0.5 μm and represents one of the major defense mechanisms that contribute to the clearing of microorganisms, pathogens, and cell debris (Engelich et al. 2001; Uribe-Querol and Rosales 2020). Monocytes express a wide range of surface receptors, such as Fc receptors for IgG (FcγRs) and complement receptors (CRs), which are very effective in the binding and internalization of opsonized particles (Mosser and Zhang 2011). Ineffective pathogen elimination is the first step in bacterial sepsis development while effective phagocytosis is a key factor in bacterial sepsis resolution (Hortova-Kohoutkova et al. 2020).

Typical features of sepsis are high fever, inflammation, immune activation as well as phagocytosis, all of which require supra-physiological energy supplies. Yet, sepsis is characterized by a clear problem in mitochondrial respiration (Wyngene et al. 2018). Glycolysis and oxidative phosphorylation (OxPhos) constitute the backbone of cellular metabolism and provide energy to the cell (McBride et al. 2020). Anaerobic glycolysis is relatively less efficient than OxPhos (Melkonian and Biochemistry 2024). Most cells in the physiological state utilize OxPhos as the metabolic pathway, but during sepsis, the metabolism of cells is reprogrammed to enhance aerobic glycolysis.

Our focus is to better understand the basis of immune dysregulation in sepsis survivors. To this end, we use a mouse model in which mice that have undergone CLP or sham surgery were allowed to recover for 4 weeks, at which time spleen cells were isolated and immune parameters evaluated. We demonstrated that in this model of polymicrobial late sepsis, glycolysis and OxPhos are increased in macrophages of CLP surviving mice for both C57BL/6 J and BALB/c strains, however, phagocytosis is only increased in C57BL/6 J CLP surviving mice. Using a single-cell RNA sequencing (scRNA-seq) approach, we further identified the subpopulations (clusters 8, 11 and 12) of CD11b^+^Ly6C^high^ cells responsible for the increased glycolysis and OxPhos in CLP surviving mice for both strains. These subpopulations are highly proliferative and express marker genes for MDSCs. Due to their high expression of Ly6C and absent expression of Ly6G at both the gene and protein levels, we classified these cells as M-MDSCs. Notably, these M-MDSC subpopulations expressed a granule protease gene signature that was more pronounced in CLP survivors compared to sham mice. Taken together, our results suggest that expanded M-MDSCs of CD11b^+^Ly6C^high^ cells may be an appropriate therapeutic target to modulate the metabolic reprogramming in sepsis survivors.

## Materials and methods

### Cecal ligation and puncture (CLP) murine model of sepsis

Male C57BL/6J and BALB/c mice that were 6 to 8 weeks old were purchased from Jackson laboratories (Bar Harbor, ME, USA) and Charles River Laboratories (Wilmington, MA, USA), respectively. The mice were housed in pathogen-free conditions at 22 °C with a 12 h light–dark cycle and had free access to a rodent diet (Lab Diet, MO, USA) and water. This study was conducted in strict accordance with recommendations in the Guide for the Care and Use of Laboratory Animals of the National Institutes of Health. The protocols were approved by the Institutional Animal Care and Use Committee and the Institutional Biosafety Committee of the Feinstein Institutes for Medical Research (Protocol number: 2009–048).

For induction of polymicrobial sepsis, animals were subjected to CLP, which induces a lethal form of peritonitis associated with 30–50% mortality (Rana et al. 2018; Cuenca et al. 2011; Rittirsch et al. 2009). CLP was performed under isoflurane anesthesia with the local anesthetic bupivacaine 0.25% (1–2 mg/kg) administered by subcutaneous injection (s.c.). Mice were given a single dosage of buprenorphine (0.05 mg/kg s.c.) before midline incision. The cecum was isolated and ligated with 4–0 silk sutures below the ileocecal valve and 1 cm from the end of the cecum, and then punctured once with a 22-G needle. Following the removal of the cecum and the extrusion of around 1 mm of feces, the abdominal muscle layer was stitched shut with 6.0 VICRYL^®^ (Ethicon, Raritan, NJ, USA) sutures, and the skin layer was stitched shut using medical-surgical clips. In sham-operated control mice, the cecum was exposed but no ligation nor puncture was performed. Both sham- and CLP-operated mice received one dose of antibiotics (imipenem/cilastatin, 0.5 mg/kg diluted in a 0.9% saline solution) as a part of the resuscitation fluid (total volume of 0.5 mL/mouse, s.c.) and a single dose of sterile saline (0.5 mL/mouse, s.c.). Mice were randomly assigned to CLP or sham treatment. Sample sizes are indicated in figure legends. Animals were checked daily for survival and assessed using the Mouse Grimace Scale twice daily for the first three days following surgery, then once daily for up to seven days.

### Flow cytometric analyses of splenocytes

Isolation, labeling, fixation, and flow cytometric analysis of mouse splenocytes were performed as previously described (Rana et al. 2018). Spleens were harvested 4 weeks after surgery, and splenocytes were isolated by crushing them with a 1 mL syringe plunger through a 70 µm strainer in ice-cold PBS without calcium or magnesium. Cell pellets were resuspended in red blood cell lysis buffer (BioLegend, San Diego, CA, USA) for 5 min at room temperature (or in 1–3 ml RBC lysis solution (5 Prime Sciences, Montreal, Canada) for 15 min for flow cytometry), washed with 2% fetal bovine serum (FBS) containing PBS, and then counted. Cells (1 × 10^6^ cells per sample) were incubated with Fc block (Rat anti-CD16/CD32, BioLegend) for 15 min at 4 °C followed by staining with phycoerythrin (PE)-Cy7-rat anti-CD11b (BD Biosciences, San Jose, CA, USA, 1:200); fluorescein isothiocyanate (FITC)-rat anti-Ly6C (BD Biosciences, 1:100); allophycocyanin (APC)-rat anti- Ly6G (BioLegend; 1:100); APC-rat anti-CD62L (BioLegend; 1:100); PerCP/Cy5.5-rat anti- CX3CR1 (BioLegend, 1:120); BV421-rat anti-CCR2 (BioLegend; 1:120); PE/Dazzle™ 594-rat anti-Mer tyrosine kinase (MERTK) (BioLegend; 1:120); BV605-rat anti-CD206 (BioLegend; 1:30); Alexa Fluor 488-rat anti-MERTK (eBioscience; 1:120); unconjugated rabbit anti-glucose transporter 1 (GLUT1) (Abcam 32551, 1:600), BV421-donkey anti-rabbit Ig (BioLegend; 1:40,000); PE-hamster anti- leukocyte-associated immunoglobulin-like receptor 1 (LAIR-1) (eBioscience; 1:60) antibodies and Fixable Viability Dye efluor506 or FVD efluor780 (Thermo Fisher Scientific) for 30 min at 4 °C. Following staining the cells were fixed in 1% paraformaldehyde and kept in the dark at 4 °C until analysis. Sample acquisition was performed using LSR Fortessa (BD Biosciences). Data were analyzed with FlowJo software (Tree Star, Inc., Ashland, OR, USA).

### Isolation of splenic monocytes, flow sorting of CD11b^+^Ly6C^high^ cells, scRNA-seq library preparation, and sequencing

Splenocytes were isolated from the spleens of sham- and CLP-operated mice C57BL/6J and BALB/c sham-operated mice 4 weeks post-surgery as described above for phenotypic and functional analyses. Monocytes were isolated from splenocyte preparations using EasySep^™^ Mouse Monocyte Isolation Kit (STEMCELL Technologies, Vancouver, Canada) according to the manufacturer’s protocol. Isolated monocytes were blocked with rat anti-mouse CD16/32 antibody (BioLegend) for 5 min at 4 °C, followed by staining with PE-Cy7-anti-CD11b and FITC-anti-Ly6C antibodies and FVD efluor506 with Total Seq^™^-B 0302 anti-mouse Hashtag 2 Antibody or TotalSeq^™^-B0303 anti-mouse Hashtag 3 Antibody or TotalSeq^™^-B0304 anti-mouse Hashtag 4 Antibody or TotalSeq^™^-B03050 anti-mouse Hashtag 5 Antibody, or TotalSeq^™^-B0306 anti-mouse Hashtag 6 Antibody (BioLegend). Five different Total Seq^™^-B antibodies were used for BALB/c and C57BL/6J, respectively, to distinguish them in the later analysis (Stoeckius et al. 2018). After combining cells from 2 sham-operated or 3 CLP surviving mice, CD11b^+^Ly6C^high^ populations were sorted using BD FACSAria IIu (BD Biosciences). The flow cytometry gating strategy used to identify the CD11b^+^Ly6C^high^ population in sham and CLP is depicted in Supplementary Fig. 1. After cell counting, cells from 2 sham-operated and 3 CLP surviving mice were combined but remained separate for C57BL/6J and BALB/c, and subsequently put onto the 10X Chromium Controller (10X Genomics, Pleasanton, CA, USA). Gel Bead-in Emulsions were generated using Chromium Next GEM Single Cell 3' Reagent Kits v3.1 (10X Genomics), following the manufacturer’s recommendations. RNA and antibody-derived tags libraries were assessed and quantitated using a High Sensitivity DNA chip (Agilent Technologies, Santa Clara, USA). Complementary DNA libraries were sequenced on Illumina HiSeq (Illumina, San Diego, CA, USA) to a depth of 26,987 reads per cell for C57BL/6J and 30,959 reads per cell for BALB/c by Azenta Life Sciences (Chelmsford, MA, USA).

### ScRNA-seq data processing and analysis

The raw sequencing data were demultiplexed into reads along with cell and unique molecular identified barcodes that were then aligned to the Mus musculus GRCm38 reference genome (Azenta Life Sciences). The generated FASTQ files were further demultiplexed using TotalSeq™ hashtags via the Cell Ranger multi pipeline by BioLegend to clarify which mouse-derived data.

The scRNA-seq dataset was processed using the Cellenics^®^ community instance (https://scp.biomage.net/) hosted by Biomage (https://biomage.net/). For the quality control process, the cut-off for mitochondrial reads was 3 median absolute deviations above the median. The linearity between the number of expressed genes per droplet and the number of Unique Molecular Identifiers (or UMIs) per droplet was examined by ‘MASS::rlm’. Outliers in the distribution of number of genes vs number of UMIs were removed by fitting a linear regression model (*P*-values between 0.00024 and 0.00072). The probability of being a doublet is calculated using ‘scDblFinder’. For each sample, the default threshold tries to minimize both the deviation in the expected number of doublets and the error of a trained classifier (Supplementary Table 1). During the process of quality control, cells with more than 5.38% mitochondrial reads and more than 0.43 probability of being a doublet were excluded (Supplementary Table 1). We then eliminated contaminating lymphocytes and obtained 23,283 individual cells with a total of 15,050 genes (Supplementary Table 1). Then, 10 datasets were integrated using the Harmony method, and principal component analysis was performed with 23 principal components. Clusters identified using the Louvain method were then visualized at a resolution of 0.8 using the dimensional reduction technique known as UMAP, to capture most of the biologically relevant information. Choosing PCs based on the cumulative variance (in this case, 80%) is a widely accepted method. By capturing 80% of the variance, we likely included most of the biologically relevant information. This approach allows for adaptability across different datasets.

Identification of marker genes and differentially expressed genes (DEGs) analyses were performed using Seurat (v5.0.1, https://satijalab.org/seurat). Predicted clusters were annotated by selecting highly ranked marker genes with p < 0.05 (up to 100 genes) and comparing those marker genes to cell marker genes from the literature; for each cluster the specific genes used for annotation and the appropriate literature citation are noted in the result section. The gene module activity score from each enriched pathway was calculated using the UCell package (v2.2.0, https://github.com/carmonalab/Ucell) on Seurat.

DEGs filtered by logFC > 0.25 and adjusted *P* values of < 0.05 were used for gene enrichment analysis against Gene Ontology Biological Process (GOBP), terms by ShinyGO 0.77 (Ge et al. 2020). UCell scores for glycolysis were calculated using genes included in the REATOME pathway (R-MMU-70171), and scores for OxPhos were calculated using genes from the KEGG pathway (mmu00190). Pathway enrichment analysis using Upregulated-DEGs (|fold|> 2 and FDR < 0.05) in CLP compared to sham (HALLMARK and KEGG).

### Lactate measurement

Monocytes were isolated from the spleen of sham-operated and CLP surviving mice 4 weeks after the surgery using EasySep™ Mouse Monocyte Isolation Kit as described above. CD11b^+^Ly6C^high^ populations were further sorted using BD FACSAria IIu (BD Biosciences). Cells (2 × 10^4^ cells per well) were cultured in X-VIVO 15 media (Lonza) overnight in a humidified 37 °C, 5% CO2 incubator. Secreted lactate levels were measured using the Lactate Colorimetric/Fluorometric Assay Kit (Biovision, Milpitas, CA, USA) following the manufacturer’s instructions. The absorbance was measured by Synergy Neo2 (BioTek, Winooski, VT, USA).

### Glycolysis measurements

Purified CD11b^+^ splenocytes or sort-purified CD11b^+^Ly6C^high^ cells (120,000 in 50 µl per well) were added to an XFp 8 well chamber (Agilent Technologies) pre-coated with Corning Cell-Tak. The chamber was centrifuged at 300 g for 1 min to promote cell adhesion and then base medium (Agilent Technologies) supplemented with 2 mM glutamine, pH 7.4 was added (130 µl per well) and the chamber incubated at 37 °C for 1 h in a non-CO2 incubator. Compounds were then added to the chamber wells, and Extracellular Acidification Rate (ECAR) was measured in real-time with Glycolysis Stress Test Kit using the Seahorse XFp Analyzer (Agilent Technologies, WAVE Desktop and Controller 2.6 Software) following manufacturer’s instructions. Oxygen consumption rate (OCR) data and OCR/ECAR ratios were obtained from the same kit. Data were normalized by cell numbers.

### RNA isolation and quantitative RT-PCR

For quantitative RT-PCR, sort-purified CD11b^+^Ly6C^high^ splenocytes were lysed with TRIzol (Invitrogen Life Technologies). DNA contaminants were removed by treatment with DNase I. Total RNA was added to RT-PCR cocktail together with primers specific for hypoxanthine guanine phosphoribosyl transferase 1 (HPRT, reference gene) or TaqMan Gene Expression Assay, Arginase 1 (Arg1, target gene) (Mm00475988m1). Forward primer sequence for HPRT is tcc tcc tca gac cgc ttt t and reverse primer sequence for HPRT is cct ggt tca tca tcg cta atc. Universal probe library #95 was used along with HPRT primers. qPCR reactions were set up according to the Light Cycler 480 RNA Master Hydrolysis Probes (#04 991 885 001) conditions reported in the Roche Diagnostics protocol (Roche Diagnostics, Indianapolis, IN, USA). All samples were run in duplicate. qPCR assays were run using the LightCycler480 (Roche Diagnostics) and the resulting data were analyzed using LightCycler 480 System software (LC480 1.5.1) (Roche Diagnostics). The mRNA level of the target genes was quantified by measuring the CT value to determine its relative expression. The results are reported using the fold change in the gene expression of the target genes relative to the reference gene (HPRT). The mean-fold change in target gene expression was calculated as 2-ΔΔCT, where DDCT = [(CT, Target–CT, reference)-(CT, Experimental–CT, reference)].

### Phagocytosis assays

Phagocytosis of sheep red blood cells (SRBC, MP Biomedicals. Santa Ana, CA, USA) was evaluated as described previously (Mosser and Zhang 2011) with slight modifications. Briefly, 2 × 10^8^ SRBCs were opsonized with anti-Sheep Red Blood Cell Stroma antibody (Sigma-Aldrich, St. Louis, MO, USA) and incubated for 1 h at room temperature with gentle rotation to resuspend the cells. SRBCs were then washed with PBS at 500 × g at room temperature for 10 min. SRBCs were then labeled with PKH26 (Sigma-Aldrich) following manufacture protocol and washed twice with RPMI medium supplemented with 10% FBS. Then 1 × 10^6^ SRBCs were incubated with 1 × 10^5^ monocytes for 1 h in a humidified 37 °C, 5% CO2 incubator.

Phagocytosis of *E. coli* was conducted using the pHrodo^™^ Red *E. coli* BioParticles^™^ Conjugates for Phagocytosis Kit according to the manufacturer’s instructions (Thermo Fisher Scientific). The particles were opsonized using BioParticles^™^ Opsonizing Reagent as per the manufacturer’s protocol (Thermo Fisher Scientific). Opsonized *E. coli* particles or particles without opsonization were then incubated with 1 × 10^5^ splenic monocytes isolated as described above at 37 °C for an hour.

After the incubation with SRBCs or *E. coli* particles, monocytes were washed, stained with PE-Cy7 anti-CD11b and FITC anti-Ly6C antibodies, fixed in buffered formalin, and analyzed by Amnis ImageStreamX Mk II Imaging Flow Cytometer (Luminex, Austin, TX, USA). Analysis of data was done through IDEAS software (Luminex). Internalized SRBCs or *E. coli* particles were measured by creating a ‘morphology’ mask for CD11b.

### Statistical analysis

Statistical analysis was performed with GraphPad Prism 10 (GraphPad Inc., La Jolla, CA, USA) using Fisher Exact Test with Bonferroni Correction, the Mann–Whitney U, Unpaired *t*-test (two groups) or one-way ANOVA (four groups) as indicated in legends. *P*-value < 0.05 was used to determine significance. For scRNA-seq data analysis, all statistical tests used in the BioConductor packages (Seurat, UCell) were performed with standard statistics tools implemented in R (version 4.23). Information about parameters and cutoffs are detailed in Methods.

## Results

### Phenotypic and functional characterization of splenic CD11b^+^Ly6C^high^ populations from CLP and sham mice utilized for scRNA-seq analyses.

**Fig. 1:**
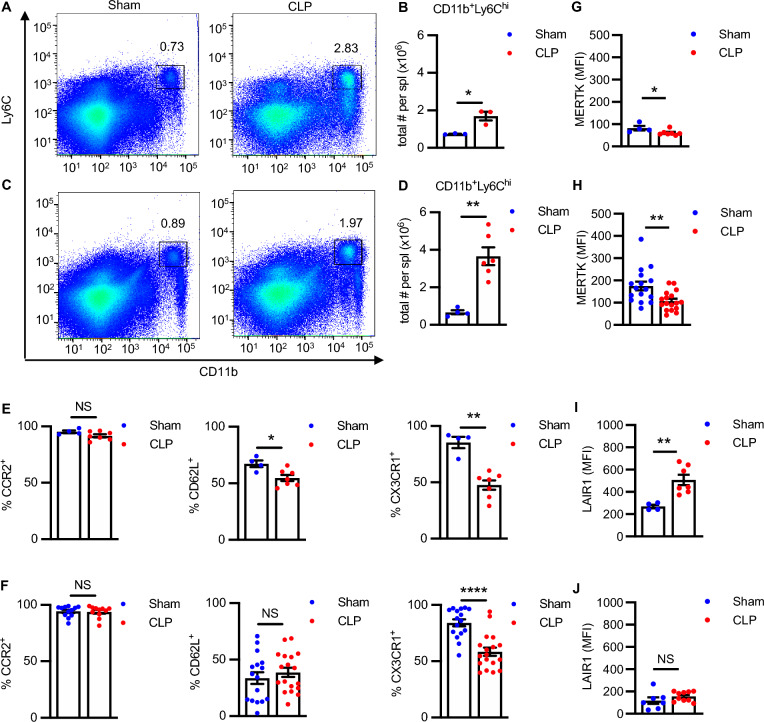
CD11b^+^Ly6C^high^ cells from sham and CLP surviving mice exhibit disease-specific and strain-specific phenotypic differences. Splenocytes from C57BL/6 J and BALB/c mice were isolated from sham and CLP surviving mice 4 weeks post-surgery, quantified by cell counting, surface stained for the indicated surface markers, and analyzed by flow cytometry. **A**–**D** Representative flow plots and quantification of CD11b^+^Ly6C^high^ populations from (**A**, **B**) C57BL/6 J (n = 3, Sham; n = 3, CLP; *P < 0.05; Unpaired t-test) and (**C**, **D**) BALB/c mice (n = 4, Sham; n = 6, CLP). **E** Percent cells positive for CCR2 (n = 4, Sham; n = 7, CLP), CD62L (n = 4, Sham; n = 7, CLP), and CX3CR1 (n = 4, Sham; n = 7 CLP) expression in CD11b^+^Ly6C^high^ populations from C57BL/6 J, and **F** percent cells positive for CCR2 (n = 12, Sham; n = 11, CLP), CD62L (n = 16, Sham; n = 18, CLP), and CX3CR1 (n = 16, Sham; n = 18 CLP) expression in CD11b^+^Ly6C^high^ populations from BALB/c mice, respectively. (**G**, **I**) MFI for MERTK (n = 4, Sham; n = 7, CLP) and LAIR-1 (n = 4, Sham; n = 7 CLP) expression in CD11b^+^Ly6C^high^ populations from C57BL/6J, and (**H**, **J**) MFI for MERTK (n = 16, Sham; n = 17, CLP) and LAIR-1 (n = 7, Sham; n = 10 CLP) expression in CD11b^+^Ly6C^high^ populations from BALB/c mice, respectively. MFI: Median fluorescence intensity. Data are presented as mean ± SEM. Sham vs. CLP **P < 0.01; ***P < 0.001; ****P < 0.0001; NS, not significant (Mann–Whitney test)

As previously reported (Valdes-Ferrer et al. 2013; Rana et al. 2018), the splenic CD11b^+^Ly6C^high^ population was markedly expanded 4 weeks after CLP surgery with significant increases in the absolute number of cells in both C57BL/6J (Fig. 1A, B) and BALB/c (Fig. 1C, D) strains. As expected, CD11b^+^Ly6C^high^ cells from sham mice expressed CCR2, CD62L, and CX3CR1, although strain-specific differences in the percentages of CD11b^+^Ly6C^high^ cells positive for these molecules were observed (Fig. 1E, F). As previously reported (Serbina and Pamer 2006), the chemokine receptor, CCR2 is required for the migration of monocytes from the bone marrow to the circulation based on reduced numbers of circulating monocytes and enhanced numbers of monocytes within the bone marrow of CCR2-/- mice. CCR2 expression did not change between sham and CLP mice (Fig. 1E, F). The adhesion molecule CD62L (L-selectin) was expressed in a subset of CD11b^+^Ly6C^high^ cells, and its expression was diminished in CLP surviving C57BL/6J (Fig. 1E), but not BALB/c, mice (Fig. 1F). The chemokine receptor CX3CR1 was expressed on the majority of CD11b^+^Ly6C^high^ cells in sham mice and decreased in CLP surviving mice for both strains (Fig. 1E, F).

We next asked whether the CD11b^+^Ly6C^high^ population has an immunosuppressive phenotype. For this purpose, we examined the expression of MERTK and LAIR-1 (Son et al. 2016). The median fluorescence intensity (MFI) of MERTK was significantly reduced on CD11b^+^Ly6C^high^ cells from CLP survivors relative to sham in both C57BL6J and BALB/c mice (Fig. 1G, H). The MFI of LAIR-1 was significantly higher in the CD11b^+^Ly6C^high^ population in CLP surviving C57BL/6J mice compared to sham mice (Fig. 1I), while no change was observed in LAIR-1 expression between sham and CLP in BALB/c mice (Fig. 1J). The expression of arginase 1 (Arg1), a marker of myeloid cells polarized toward an immunosuppressive phenotype, was also shown to be significantly increased in BALB/c CD11b^+^Ly6C^high^ cells from CLP survivors relative to sham (Supplementary Fig. 3F). Taken together, these results suggest that CD11b^+^Ly6C^high^ cells express immunosuppressive markers that might contribute to its immunosuppressive phenotype.

**Fig. 2:**
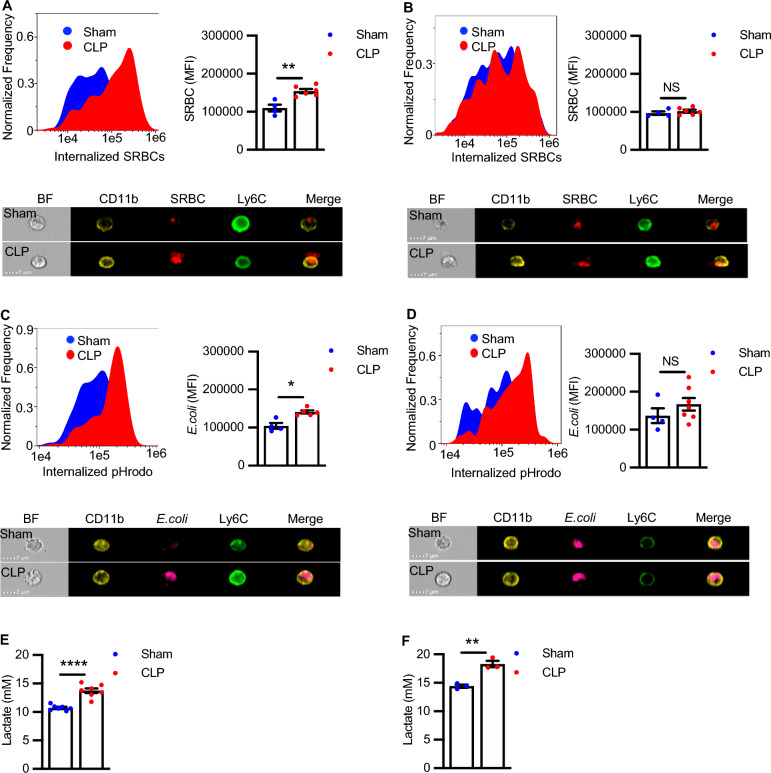
CD11b^+^Ly6C^high^ cells from sham and CLP surviving mice exhibit disease-specific and strain-specific differences in phagocytosis and glycolysis. Splenocytes from Sham and CLP (**A**, **C**, **E**) C57BL/6J and (**B**, **D**, **F**) BALB/c mice were isolated 4 weeks post-surgery, and phagocytic and glycolytic function were assessed. **A**, **B **Representative histogram plots and mean fluorescent intensity (MFI) of internalized SRBCs in (**A**) C57BL/6J (n = 4, Sham; n = 6, CLP) and (**B**) BALB/c mice (n = 4 Sham, n = 6 CLP). Representative histogram plots and MFI of internalized *E. coli* particles in (**C**) C57BL/6J (n = 4, Sham; n = 5 CLP), and (**D**) BALB/c (n = 4, Sham; n = 7 CLP) mice. Sham vs. CLP *P < 0.05; **P < 0.01; NS, not significant (Mann–Whitney test). Representative image of internalized SRBCs for one CD11b^+^Ly6C^high^ cell from sham and one CD11b^+^Ly6C^high^ cell from CLP in (**A**) C57BL/6J and (**B**) BALB/c mice, respectively. Representative image of internalized *E.coli* particles for one CD11b^+^Ly6C^high^ cell from sham and one CD11b^+^Ly6C^high^ cell from CLP in (**C**) C57BL/6J and (**D**) BALB/c mice, respectively. Lactate release in cultured CD11b^+^Ly6C^high^ cells isolated from sham and CLP (**E**) C57BL/6J (n = 7, Sham; n = 7, CLP) (**F**) BALB/c (n = 3, Sham; n = 3 CLP) mice. Results are presented mean ± SEM. Sham vs. CLP **P < 0.01, ****P < 0.0001 (Unpaired t-test)

Functional heterogeneity was observed in splenic CD11b^+^Ly6C^high^ cells as well. Since phagocytosis is a crucial element in the immunological defense against pathogens (Guilliams et al. 2018), we examined the phagocytic capacity of CD11b^+^Ly6C^high^ cells. The ImageStream gating strategy and cell masking for phagocytosis of SRBCs and *E. coli* is depicted in Supplementary Fig. 2A–C. CD11b^+^Ly6C^high^ cells from both C57BL/6J and BALB/c sham mice exhibited robust and similar levels of phagocytosis, but the effects of sepsis on phagocytosis differed between the two strains. Four weeks post-surgery, CD11b^+^Ly6C^high^ cells from CLP surviving C57BL/6J mice exhibited enhanced phagocytosis of both opsonized SRBCs and *E. coli* particles (Fig. 2A, C), while no increases were observed in BALB/c mice (Fig. 2B, D). Notably, the increased phagocytosis of *E. coli* particles was abolished when the particles were not opsonized by polyclonal IgG antibodies against *E. coli* demonstrating enhanced Fc receptor-mediated phagocytosis in CLP surviving C57BL/6J (Supplementary Fig. 2D).

Infection alters myeloid cell metabolism, skewing it towards glycolysis. To see if the alteration persisted in sepsis-surviving mice, we measured lactate release from CD11b^+^Ly6C^high^ cells isolated from sham and CLP mice as an indicator of glycolysis. Lactate release was significantly higher in CD11b^+^Ly6C^high^ cells from CLP mice compared to sham mice in both C57BL/6J (Fig. 2E) and BALB/c mice (Fig. 2F). Glycolysis is also measured through ECAR of the medium, which is predominately from the excretion of lactate. We performed a glycolysis stress assay in sham and CLP CD11b^+^Ly6C^high^ cells from BALB/c mice and observed that ECAR and the average rate of glycolysis were increased in CLP as compared to sham mice (Supplementary Fig. 3A, B), consistent with our results with lactate release (Fig. 2F). Energy maps plotting ECAR versus OCR at baseline and after the addition of glucose (Supplementary Fig. 3C) show that ECAR and OCR are both increased in BALB/c CLP mice as compared to sham mice, indicating that at least a portion of the CD11b^+^Ly6C^high^ cells from CLP survivors upregulate both glycolysis and oxidative phosphorylation pathways to supply their energy needs**.** Of note, the OCR/ECAR ratio, a qualitative measurement of the relative utilization of mitochondrial (oxidative) versus glycolytic pathways for energy production, was lower in BALB/c CLP CD11b^+^Ly6C^high^ cells (1.3) relative to sham (1.9) after the addition of glucose, indicating a more glycolytic bioenergetic balance. The expression of the GLUT1, which facilitates the transport of glucose across the plasma membranes, and glucose uptake itself, were both significantly higher in BALB/c CLP mice as compared to sham mice (Supplementary Fig. 3D, E). Taken together, although the CD11b^+^Ly6C^high^ population express markers of myeloid cells, the inherent phenotypic and functional heterogeneity we observe suggests that the population is comprised of multiple subsets that may differ functionally. To address the phenotypic and functional complexity in the CD11b^+^Ly6C^high^ cell population we subjected these cells to scRNA-seq analysis.

### ScRNA-seq reveals heterogeneity within CD11b^+^Ly6C^high^ monocytes

**Fig. 3:**
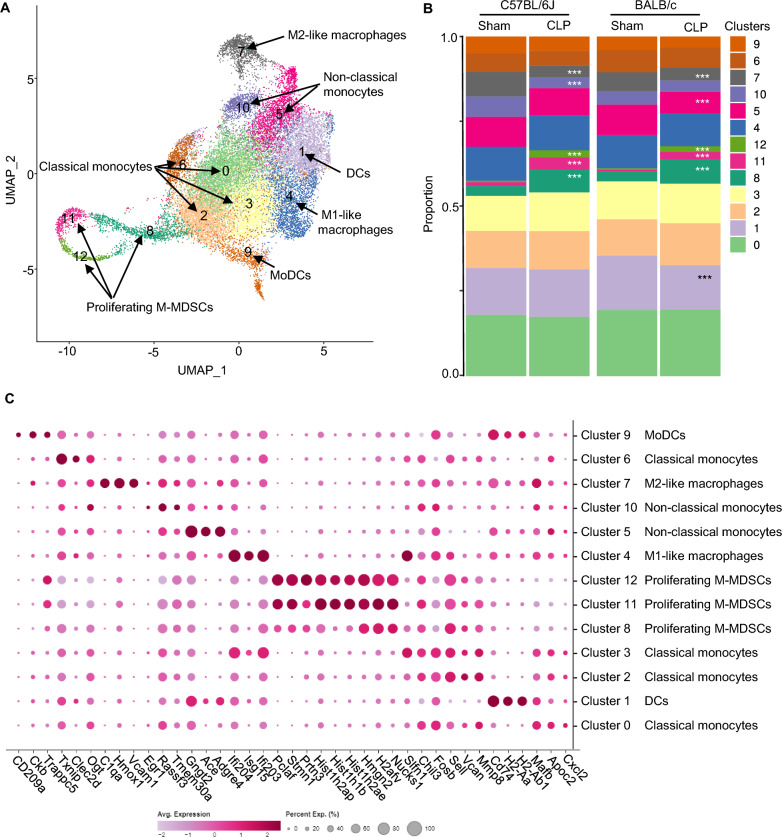
CD11b^+^Ly6C^high^ monocytes clustering in single-cell RNA-seq shows diverse phenotypes. **A** UMAP plot of single-cell CD11b^+^Ly6C^high^ cells from sham and sepsis-surviving mouse (CLP) spleen. Cells are colored by unsupervised clustering of all four groups (Sham and CLP of both C56BL/6J and BALB/c strains) (sham, n = 2) and (CLP, n = 3). Corresponding (predicting) cell type annotated based on known cell-type-specific marker genes: classical monocytes (clusters 0, 2, 3, 6), dendritic-like cells (cluster 1), M1-like macrophages (cluster 4), non-classical monocytes (clusters 5, 10), M2-like macrophage (cluster 7), monocyte-derived dendritic-like cells (cluster 9), proliferating monocytic myeloid-derived suppressor cells (M-MDSCs) (clusters 8, 11, 12). **B** Cell-type composition plots (Frequency maps) of 13 clusters in each group. Sham vs. CLP ***P < 0.001 (Fisher Exact Test with the Bonferroni adjustment). **C** Dot plot of genes defining the 13 clusters of CD11b^+^Ly6C^high^ cells from sham and sepsis-surviving mice of both C56BL/6J and BALB/c strains. The percentage of gene expression in all the cells of a specific cluster is represented by the size of the dot. The color reflects the level of expression of the gene

Splenic CD11b^+^Ly6C^high^ cells from C57BL/6J and BALB/c were obtained as described in methods and subjected to scRNA-seq analysis. Unsupervised clustering that is based on principal components that explains 80% of total variance resulted in distribution of CD11b^+^Ly6C^high^ cells into thirteen discrete clusters (Fig. 3A). Four weeks after the surgery, CD11b^+^Ly6C^high^ cells from both C57BL/6J and BALB/c mice exhibited similar clusters despite their distinct genetic backgrounds, although several strain-specific differences in the proportions of the 13 individual clusters were apparent in the sham and CLP cohorts of the two strains (Fig. 3A–B, Supplementary Tables 2 and 3 [subset frequencies and statistics, respectively]). Frequency plots for cell sets also showed significant differences between sham and CLP CD11b^+^Ly6C^high^ cells for a subset of clusters. Clusters 8, 11 and 12 were expanded and cluster 7 was decreased in CLP surviving mice as compared to sham mice in both strains (Fig. 3B, Supplementary Table 2). There were also strain-specific differences: cluster 10 was decreased in C57BL/6J (but not BALB/c) CLP survivors, while clusters 1 and 5 were decreased in BALB/c (but not C57BL/6J) CLP survivors (Fig. 3B, Supplementary Tables 2 and 3). Next, clusters were defined using marker genes (Fig. 3A, C, Supplementary Table 4, Supplementary Fig. 4). Clusters 0, 2, 3, and 6 were annotated as classical monocytes based on typical markers, *Fn1, F13a1, Sell, Ccr2, MMP8, Chil3, Ly6c2*, and *Cd14* expression (Hey et al. 2019; McGinnis et al. 2023) (Fig. 3A, C, Supplementary Table 4, Supplementary Fig. 4). Cluster 1 and 9 expressed MHC II genes (*H2-Ab1, H2-Aa, H2-Eb1*), *Cd74, Tmem176a/b* (Fig. 3A, C, Supplementary Table 4, Supplementary Fig. 4), and both clusters were enriched in GO regarding antigen presentation (*data not shown*). Cluster 1 expressed *Ciita* and *Batf3* genes and were annotated as DC-like cells, while cluster 9 expressed *Cd209a* and were annotated as MoDC-like cells (Menezes et al. 2016; Anderson et al. 2017; Lancien et al. 2021) (Fig. 3A, Supplementary Table 4, Supplementary Fig. 4). Cluster 4 was enriched in interferon-stimulated genes (*Isg15, Ifi204, Ifit3, Irf7, Oasl2, Rasd2, Cxcl10*) and were annotated as M1-like macrophages/ISG^+^-stimulated macrophages (Subramani et al. 2023; Ma et al. 2018) (Fig. 3A, Supplementary Table 4, Supplementary Fig. 4). Clusters 5 and 10 expressed *Nr4a1, Pou2f2, Ace, Ear2, Eno3, Gngt2, Cd300e, Cd36, Fcgr4, Dusp, Spn, Adgre4, Itgal* and were annotated as non-classical monocytes (Hey et al. 2019; McGinnis et al. 2023) (Fig. 3A, Supplementary Table 4, Supplementary Fig. 4), as previously described (Mildner et al. 2017). Complement C1q expressing-cluster 7 (*C1qb, C1qc, C1qa, Slc40a1, Mrc1, Hmox1, Vcam1, Cd163*) was designated as M2-like macrophages (Quero et al. 2017; Spivia et al. 2014) (Fig. 3A, Supplementary Table 4, Supplementary Fig. 4). Clusters 8, 11, and 12 were characterized by proliferation-associated genes including *Mki67*, *Stmn1, Ube2c, Cenpf, Top2a, Hist1h2ap, Hist1h1b, Hist1h2ae, Pclaf, Birc5* and expression of *S100a8/9, Hmgb1, Hmgn2, Ppia, Tubb5, Ngp, Ltf,* and *Wfdc21* (Fig. 3A, Supplementary Table 4, Supplementary Fig. 4). These clusters of immature cells were annotated as M-MDSCs based on their gene expression profile and Ly6C^high^ surface expression and absence of Ly6G (DaSilva et al. 2021; Dietrich et al. 2022; Sanchez-Pino et al. 2021). Of note, Clusters 8, 11, and 12 strongly expressed genes encoding granule proteases including *Elane*, *Prtn3*, and *Lcn2* typically associated with azurophilic and secondary granules (Supplementary Fig. 5) (Sekheri et al. 2021; Sheshachalam et al. 2014). As noted above, several clusters are similar, as shown in Fig. 3A and C, and express similar defining genes (7 groups). This observation suggests that some clusters represent subtypes of the same cell type and may relate to differences in activation state. When similar clusters were combined together leading to 7 clusters and frequencies were recalculated, the subtle differences between strains were lost, with the exception of DCs, which remained significantly lower in CLP survivors relative to sham in BALB/c, but not in C57BL/6J (Supplementary Table 5). Collectively, scRNA-seq reveals differentiation pathways that produce classical monocytes, non-classical monocytes, DC-like cells, M1- or M2-like macrophages, MoDC-like cells and proliferating M-MDSCs, confirming heterogeneity in the splenic CD11b^+^Ly6C^high^ population.

### Glycolysis, OxPhos, and cell cycle pathways and genes encoding granule proteases are upregulated in expanded M-MDSCs of CD11b^+^Ly6C^high^ cells in sepsis-surviving mice.

**Fig. 4:**
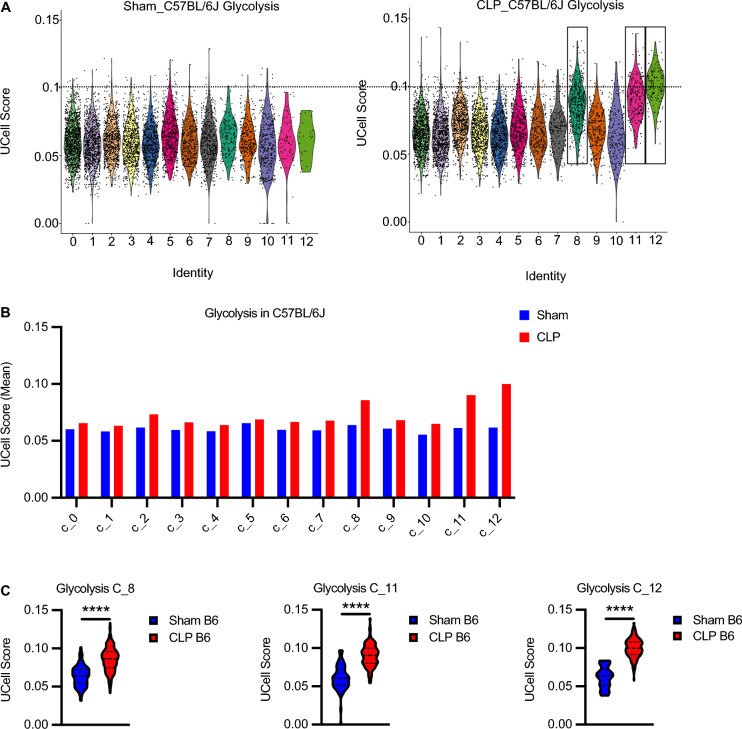
The expression of genes of the glycolysis and cell cycle pathway in clusters 8, 11 and 12 in sepsis-surviving mice are upregulated. **A** Violin plots and **B** bar graphs showing UCell score of glycolysis in Sham (n = 2) and CLP (n = 3) in C57BL/6J. UCell scores for glycolysis were calculated using genes from the REATOME pathway (R-MMU-70171 and Table S6). **C** Clusters 8, 11 and 12 in CLP show higher levels of glycolysis genes than sham. Sham vs. CLP ****P < 0.0001 (Mann–Whitney test)

**Fig. 5:**
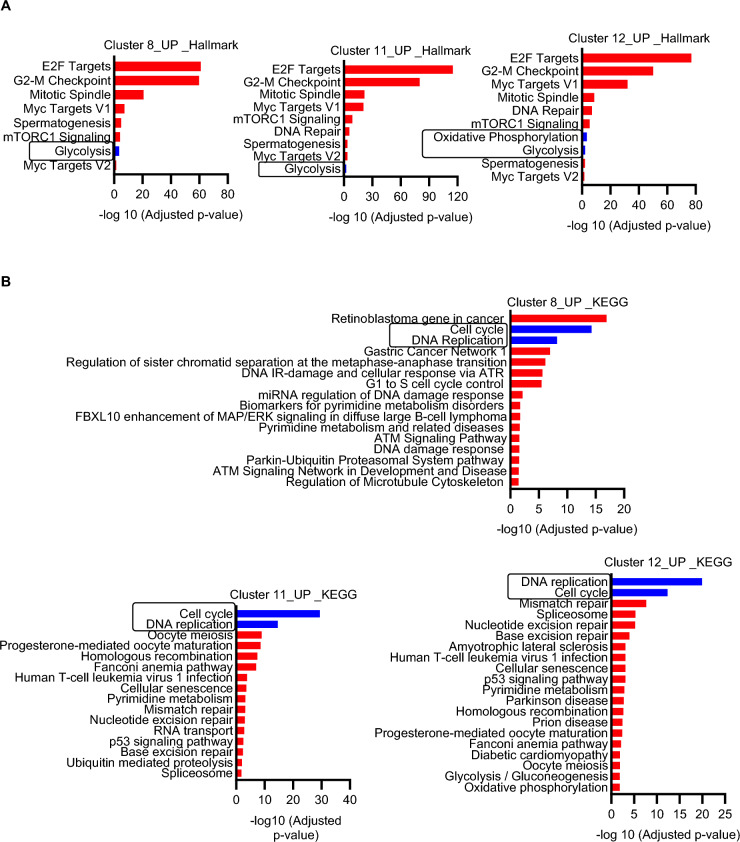
Pathway analysis showed that DEGs upregulated in CLP were significantly enriched in the glycolysis, OxPhos, and DNA replication and cell cycle. **A** The glycolysis pathway is upregulated (highlighted in blue) in clusters 8, 11 and 12 in CLP (Hallmark) and the OxPhos pathway is upregulated (highlighted in blue) in cluster 12 in CLP (Hallmark). **B** Proliferation-related genes are upregulated (highlighted in blue) in clusters 8, 11 and 12 in CLP (KEGG pathway; mmu00190). The x-axis is adjusted *P*-value (< 0.05) log transformed in GraphPad prism

DEGs and gene set enrichment analysis further showed enhanced cell division in CLP surviving mice compared to sham mice (Supplementary Table 6). Recent studies have revealed that rewiring cellular metabolism is a crucial step for the adaptation of the innate immune system (Arts et al. 2016). Therefore, we next investigated metabolic changes induced by sepsis using UCell scores for glycolysis or OxPhos pathway. UCell score calculates individual cell scores for each signature based on the Mann–Whitney U statistical analysis. UCell score analysis was performed with gene signatures well known for glycolysis and OxPhos (Supplementary Table 7). Clusters 8, 11 and 12 in CLP C57BL/6J mice (Fig. 4 A–C) and cluster 8 in CLP BALB/c mice (Supplementary Fig. 6A–C) showed the highest gene signature UCell score for glycolysis. Similarly, UCell score for OxPhos was up in nearly all clusters but predominately observed in cluster 8, 11 and 12 in CLP C57BL/6J (Supplementary Fig. 7A–C) and BALB/c mice (Supplementary Fig. 7D–F) when compared to sham mice. Pathway analysis was performed on upregulated DEGs genes with fold enrichment 2.0 and FDR p-value < 0.05. The results of the Hallmark and KEGG pathway analysis showed that the upregulated DEGs were significantly enriched in glycolysis (Hallmark; Fig. 5A) and DNA replication and cell cycle (KEGG; Fig. 5B) in clusters 8, 11 and 12 in CLP C57BL/6J mice. The upregulated DEGs were also significantly enriched in the OxPhos pathway in cluster 12 in CLP C57BL/6J mice. (Fig. 5A). Thus, there was concordance between the data from scRNA-seq and functional metabolic profiling. The expression levels of genes encoding granule proteases including *Elane*, *Prtn3*, *Lcn2* and *Fcnb* were significantly higher in clusters 8, 11 and 12 from CLP surviving C57BL/6J mice compared to sham-operated mice as well (Supplementary Fig. 8). A similar trend of increased granule protease expression was observed in clusters 8 and 11, but not cluster 12, in CLP surviving BALB/c mice although the difference only reached statistical significance for all proteases in cluster 8 (Supplementary Fig. 9).

Collectively, sepsis-surviving mice showed an expansion of CD11b^+^Ly6C^high^ cells; CLP expands M-MDSCs and induces their metabolic reprogramming.

### Increased phagocytosis is reflected in the scRNA-seq of CLP surviving C57BL/6J mice

**Fig. 6:**
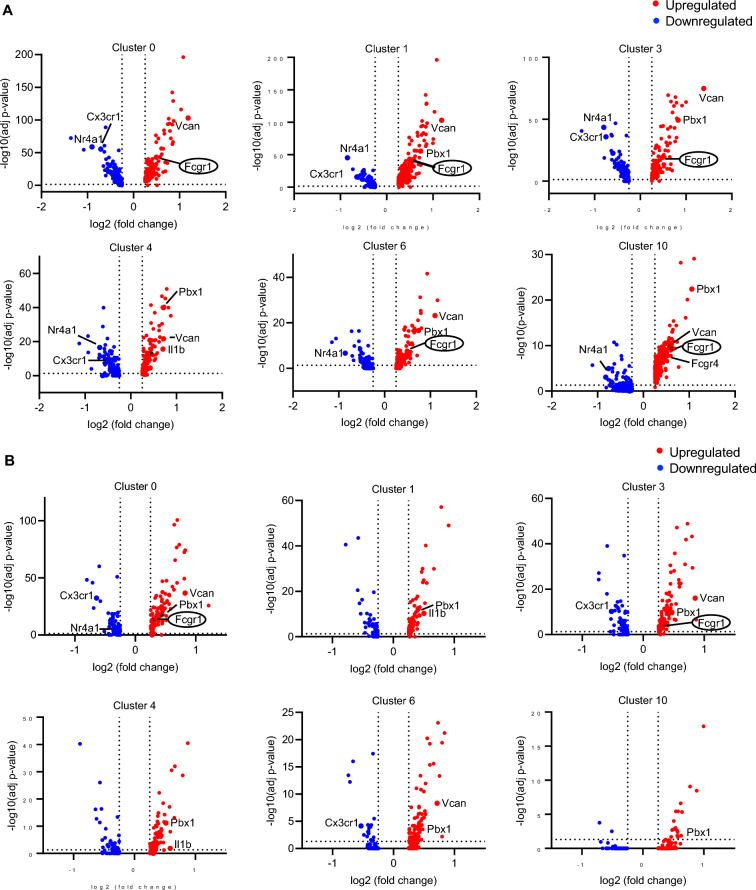
Fc gamma receptor-dependent enhancement of phagocytosis in sepsis- surviving C57BL/6J mice. Volcano plots of the DEGs (|fold|> 2 and FDR < 0.05) in CLP compared to sham in C57BL/6J mice (**A**) and in BALB/c mice (**B**). The y-axis is the -log10-based adjusted p-value, and the x-axis is the log fold change (logFC). Upregulated genes in CLP are colored red, down-regulated genes in CLP are colored blue, and genes of interest are labeled

**Fig. 7:**
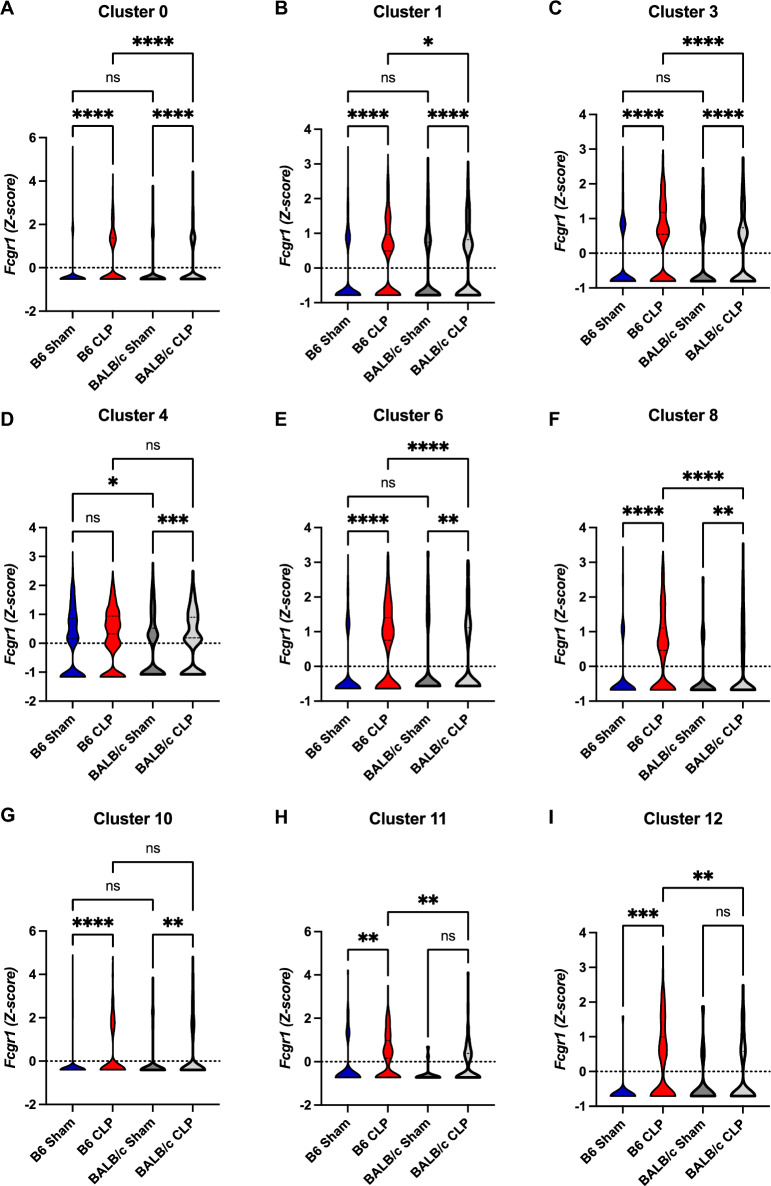
Upregulation of Fc gamma receptors in sepsis- surviving C57BL/6J mice. **A**–**F** Violin plots of Fcgr1 indicated clusters. C57BL/6J Sham vs. C57BL/6J CLP vs. BALB/c Sham vs. BALB/c CLP. *P < 0.05, **P < 0. 01, ***P < 0.001,****P < 0.0001 (one-way ANOVA)

**Fig. 8:**
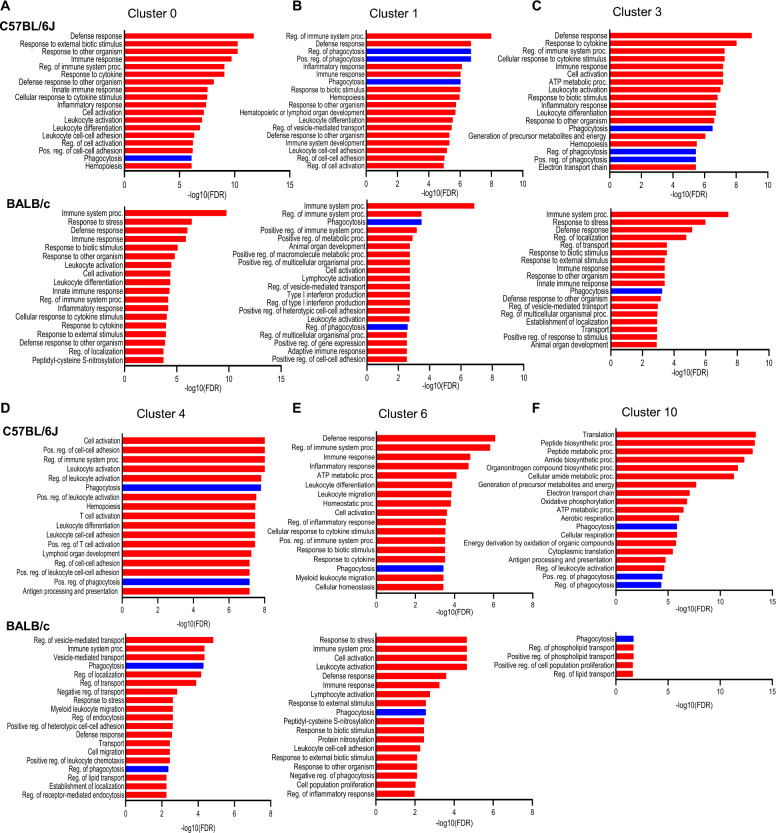
Phagocytic pathways are upregulated in various clusters of CD11b^+^Ly6C^high^ cells in C57BL/6J sepsis-surviving mice. **A**–**F** GOBP enriched by upregulated DEG in each cluster. Phagocytosis and phagocytosis-related pathways are highlighted in blue

Phagocytosis of opsonized SRBCs and *E. coli* particles was increased in CLP mice. As it is Fc gamma receptor-dependent, we asked which clusters express FcγRI (*Fcgr1*)*.* Volcano plots revealed that among DEGs between sham and CLP mice, FcγRI was upregulated in several clusters (0, 1, 3, 6 and 10), particularly in classical monocyte clusters (0, 3, and 6) of C57BL/6J mice (Fig. 6). There was less statistical significance in BALB/c. Further, the expression levels of FcγRI were significantly increased in CLP surviving compared to sham-operated C57BL/6J mice in these clusters (Fig. 7). Notably, the expression levels of FcγRI were significantly increased in CLP surviving C57BL/6J compared to BALB/c mice (Fig. 7, Supplementary Table 6, 8). Gene enrichment analysis also confirmed the augmentation of phagocytosis-related GOs in clusters 0, 1, 3, 6 and 10 with greater statistical significance compared to BALB/c (Fig. 8). These data suggest that classical monocytes in CLP C57BL/6J mice have enhanced phagocytic capacity that may be mediated by FcγRI.

## Discussion

We provided evidence in this study that the splenic CD11b^+^Ly6C^high^ myeloid population expanded in mice surviving sepsis contributes to immunosuppression and is attributable, at least in part, to metabolic reprogramming. We demonstrated that the experience of sepsis affects the phenotypic and functional responses of CD11b^+^Ly6C^high^ cells in C57BL/6J and BALB/c mice. Differences between sham- and CLP-operated mice were observed, some of which were common between strains and others that differed. ScRNA-seq of CD11b^+^Ly6C^high^ cells identified thirteen distinct clusters that are observed in both strains including some that transcriptionally align with classical monocytes, as well as 9 clusters with signature genes of non-classical monocytes, DCs, MoDCs, macrophages or proliferating M-MDSCs. In particular, the expansion of proliferating M-MDSCs, which were metabolically rewired and associated with neutrophil granule protease genes, was prominent in sepsis-surviving mice in both strains.

As previously reported (Rana et al. 2018), a marked expansion of the CD11b^+^Ly6C^high^ myeloid population occurs in the spleen of CLP mice. We have explored the mechanisms that are responsible for this expansion. The chemokine receptor CCR2 controls the release of CD11b^+^Ly6C^high^ monocytes from the bone marrow and their recruitment to the sites of infection or inflammation (Li et al. 2008). In both strains, the levels of CCR2 on splenic CD11b^+^Ly6C^high^ cells did not differ between CLP mice and sham-operated mice. Another chemokine receptor, CX3CR1, reduces the motility of Ly6C^high^ monocytes in the bone marrow and thereby controls their release (Li et al. 2008). Reduced expression of CX3CR1 was observed on CD11b^+^Ly6C^high^ monocytes in CLP surviving mice in both strains suggesting that these cells are less likely to be retained in the bone marrow thereby facilitating their recruitment into the spleen. In line with our study, CX3CR1 is reduced in lung Ly6C^high^ monocytes of CLP mice (Baudesson de Chanville et al. 2020) due to its increased internalization (Ge et al. 2016). This may partially account for the higher numbers of CD11b^+^Ly6C^high^ monocytes that are observed in sepsis-surviving mice in both strains.

LAIR-1 expression on monocytes is upregulated during an inflammatory response and acts as an immune-inhibitory receptor implicated in immune suppression (Laethem et al. 2022). MERTK is involved in efferocytosis and has been shown to be cleaved by a disintegrin and metalloprotease 17 (ADAM17) resulting in its reduced surface expression under inflammatory conditions (Cai et al. 2016). MERTK expression was significantly downregulated in CD11b^+^Ly6C^high^ monocytes in CLP mice of both strains, although the decrease was more pronounced in BALB/c. In contrast to MERTK, which was regulated similarly in the two mouse strains, LAIR-1 expression was differentially regulated in C57BL/6J and BALB/c in the context of sepsis, with LAIR-1 expression significantly upregulated only in CLP C57BL/6 CD11b^+^Ly6C^high^ monocytes. While these changes are consistent with the notion that their expression increases and decreases, respectively, under inflammatory milieu, the subtle differences in how these two proteins are regulated between the two strains of mice suggest that the mechanisms contributing to immunosuppression in sepsis survivors may be distinct in the two strains. Alternatively, these differences may reflect baseline differences in the proportions of individual clusters in the two strains.

The phagocytic capacity of CD11b^+^Ly6C^high^ cells after CLP surgery is affected by the timing at which this population is analyzed, its origins, and the experimental design, such as in vivo or ex vivo (Baudesson de Chanville et al. 2020; Ocuin et al. 2011). Opsonic receptors, mainly FcRs, bind antibodies coating the pathogen surface (Hortova-Kohoutkova et al. 2020). Seminal experiments using IgG-opsonized SRBCs or *E.coli* demonstrated that their internalization is mediated exclusively through FcγR-mediated pathways by splenic macrophages (Bournazos et al. 2016). It has been reported that in the acute phase of sepsis patients have a worse prognosis and shorter survival with reduced expression of FcγRI (CD64) and impaired phagocytic activity (Danikas et al. 2008). In a study of patients with septic shock exhibiting compensatory anti-inflammatory response syndrome (CARS) monocyte phagocytic activity was notably reduced contributing to functional decline and immune paralysis (Xu et al. 2012). The previous two studies involved patients in acute sepsis or septic shock. In our study of mice 4 weeks after CLP-surgery (sepsis survivors) or sham surgery, we observed increased phagocytosis of IgG-opsonized *E. coli* or SRBCs by CD11b^+^Ly6C^high^ monocytes in C57BL/6J CLP mice. ScRNA-seq likewise revealed that clusters 0, 3, and 6 of classical monocytes showed increased expression of FcγRI in CLP mice compared to sham mice; this correlated with enhanced phagocytic activity. Interestingly, in line with our study, FcγR expression in association with CD89 (FcαRI) has been shown to be upregulated on blood phagocytes following bacterial infections and mediates increased phagocytosis (Tymowski et al. 2019; Chiamolera et al. 2001) indicating the crucial role of FcRs in mediating host defense against sepsis. Moreover, we previously reported that CLP mice exhibited progressively increased bacterial clearance 4 weeks post-surgery (Rana et al. 2021), suggesting efficient phagocytosis. Strain-specific differences also should be considered for phagocytosis, as we failed to see the enhancement of phagocytosis in BALB/c mice (*data not shown*). Monocyte phagocytosis has a key role during sepsis resolution and therefore differences in sepsis-induced enhancement of phagocytosis between C57BL/6J and BALB/c may reflect differences in the ability of the two strains to mount an antipathogen response and is consistent with their decreased survival in the CLP model (Watanabe et al. 2004).

CD11b^+^Ly6C^high^ classical monocytes have been reported to be pro-inflammatory, but there is a functional plasticity in this cell population. A study using an experimental model of autoimmune encephalomyelitis revealed that CD11b^+^Ly6C^high^ cells, which were markedly increased in the spleen during disease development, were highly suppressive for activated T cells (Zhu et al. 2007). In a pristane-induced lupus model, CD11b^+^Ly6C^high^ monocytes were mobilized, expanded to M-MDSCs, and shown to be capable of inhibiting T cell proliferation ex vivo (Ma et al. 2016). Another study reported that during staphylococcus aureus biofilm infection, a heterogenous population of CD11b^high^ Ly6G^+^ Ly6C^+^ MDSCs proliferated locally at the site of infection and suppressed T cell proliferation (Heim et al. 2018). These studies suggest that CD11b^+^Ly6C^high^ populations are heterogenous with immunosuppressive functions. Therefore, it will be informative to perform suppression assays using CD11b^+^Ly6C^high^ cells in coculture with anti-CD3/CD28-stimulated CD4^+^ T cells to determine whether splenic CD11b^+^Ly6C^high^ M-MDSCs populations in CLP surviving mice are able to inhibit proliferation of CD4^+^ T cells.

Advances in single-cell multi-omics technologies have redefined how we interpret hematopoiesis (Abbas et al. 2018), especially monopoiesis (Guilliams et al. 2018; Weinreb et al. 2020), and reveal monocyte heterogeneity in steady-state as well as sepsis (Yanez et al. 2017; Villani et al. 2017). To observe the continuing blood regeneration in bone marrow in the steady-state, a recent study tagged hematopoietic progenitor cells with DNA barcodes (Weinreb et al. 2020). This revealed two separate pathways for DC-like or neutrophil-like monocyte differentiation resulting in different gene expression patterns in mature cells in bone marrow (Weinreb et al. 2020). Inflammatory conditions induce emergency monopoiesis leading to functionally distinct monocyte subsets. These emergent monopoietic and phenotypic alterations during infections can have long-lasting effects characterized by a change in cell fate resulting in increased numbers of specific monocyte subsets and different activation states weeks after pathogen clearance (Guilliams et al. 2018). By utilizing scRNA-seq approach, Ly6C^high^ monocytes have been shown to be metabolically reprogrammed in the blood following an inflammatory stimulus; this facilitates their differentiation into M2-like macrophages (Purvis et al. 2021). A recent single-cell transcriptome study identified HLA-DR^low^S100A^high^ monocytes with immunosuppressive function in late (up to 72 h) sepsis (Yao et al. 2023). Another scRNA-seq pilot study revealed three MDSC subset clusters -granulocytic (G-), monocytic (M-), and early (E-) MDSCs in late sepsis patients (Darden et al. 2021). Our transcriptional profiling approach revealed that clusters within CD11b^+^Ly6C^high^ population exhibit signature genes of M-MDSCs. These clusters are expanded in CLP and have higher expression of S100A8/9 as evident by DEGs analysis in the spleen of sepsis-surviving mice. LPS has been demonstrated to induce the mobilization of IMCs from bone marrow to peripheral tissues in an emergency myelopoiesis manner where IMCs proliferate and become MDSCs through activation at extramedullary sites, especially the spleen (Millrud et al. 2017). Pathway analysis displayed upregulated expression of several cell cycle genes for CD11b^+^Ly6C^high^-derived M-MDSCs in CLP mice indicating that M-MDSCs were dividing within the spleen. Our results support the notion that extramedullary myelopoiesis and increased spleen size seen in sepsis-surviving mice are due, at least in part, to local proliferation of CD11b^+^Ly6C^high^-derived M-MDSCs.

A scRNA-seq analysis Ly6C^high^ monocyte populations isolated from mouse bone marrow identified a cluster of cells enriched in neutrophil granule protein genes, which the authors termed “neutrophil-like” monocytes (Yanez et al. 2017). Under steady state conditions these cells differentiated from granulocyte macrophage progenitors (GMPs), and following injection of LPS to mimic pathogen challenge adoptively transferred GMPs differentiated into these “neutrophil-like” monocytes in vivo (Yanez et al. 2017). Subsequent studies report that this population is released during inflammation-induced emergency monopoiesis (Ikeda et al. 2023; Gudenschwager Basso et al. 2024). Whether this population is present in the CLP model of polymicrobial sepsis or in human sepsis is currently unknown. Of note, we observed strong expression of genes encoding granule proteins (MPO, Lcn2, elastase and prtn3) typically expressed by neutrophils in the three M-MDSC clusters that are present at higher frequencies in CLP mice. These granules can fuse with nascent phagosome, releasing their cargo into vacuole to kill ingested pathogens or with plasma membrane, resulting in secretion of granule proteins extracellularly (degranulation) (Othman et al. 2022). Upon their release, granule proteins determine mode of cell death of neutrophils e.g. NETosis, or necroptosis (Othman et al. 2022).

While these clusters share a number of similarities with the “neutrophil-like” monocytes identified by Yanez et al. (Yanez et al. 2017), including strong expression of genes encoding granule proteases as well as S100A8, S100A9, the relationship between these populations is unclear as their gene expression profiles are not fully aligned. Whether these populations derive from a common progenitor and how they relate to one another is an area of future study, but it is interesting to note that there are reports that M-MDSCs in multiple cancers including glioblastoma, lung, and head and neck cancers, exhibit a neutrophil-like monocyte transcriptional state (Wiencke et al. 2023; Soler et al. 2017; Zilionis et al. 2019; Abdelfattah et al. 2022). The current report is the first time this has been described in the context of sepsis.

The increased production of lactate in sepsis is thought to be the result of increased glycolysis activity (aerobic glycolysis a.k.a Warburg effect), a well-known phenomenon in sepsis involves a shift in metabolism away from OxPhos towards aerobic glycolysis to power proliferation and biosynthesis (Wyngene et al. 2018; Preau et al. 2021). UCell score analysis revealed that only the three M-MDSC clusters showed elevated glycolysis-related transcripts, although basal glycolysis of the CD11b^+^Ly6C^high^ population as a whole was enhanced in CLP surviving mice as shown by increased lactate release and GLUT1 expression. This suggests that M-MDSCs are rewired metabolically to meet the increased energy demand to support rapid proliferation. It is important to note that the lactate produced as a consequence of enhanced glycolysis in sepsis-surviving mice may itself have immunosuppressive effects as it has been shown to trigger metabolic reprogramming of immune cells, including monocytes and macrophages, and promoting an anti-inflammatory, tolerogenic microenvironment (Nolt et al. 2018). All clusters of CD11b^+^Ly6C^high^ including M-MDSCs showed upregulation of OxPhos-related genes, confirming another recent report (Purvis et al. 2021) and suggesting that cells can simultaneously upregulate both pathways.

This study has several limitations. Firstly, we focused exclusively on CD11b^+^Ly6C^high^ cells and therefore are unable to relate our findings to other splenic myeloid populations. That said, this approach enabled us to perform deep-sequencing and obtain a higher resolution. Secondly, since CLP is a model of polymicrobial sepsis, the specific pathogens responsible for the phenotypic changes observed are unknown. However, this model is clinically relevant, and the findings from this study have important clinical implications. Thirdly, we evaluated CD11b^+^Ly6C^high^ cells at a single time point (4 weeks post-surgery) and therefore the study lacks temporal information. Fourthly, we analyzed CD11b^+^Ly6C^high^ cells from mice that survive an episode of sepsis and additional studies are required to determine how our findings relate to monocyte populations present in patients who survive a septic episode. Lastly, we used scRNA-seq to identify cell clusters within the parent CD11b^+^Ly6C^high^ population and have not yet purified each of the thirteen CD11b^+^Ly6C^high^ subpopulations and evaluated for functionality, including suppressive activity. This will be an area of future studies.

In conclusion, we have revealed the diversity of the splenic CD11b^+^Ly6C^high^ monocyte population in sham-operated and CLP mice using scRNA-seq analysis. In sepsis-surviving mice, proliferating M-MDSC subpopulations of CD11b^+^Ly6C^high^ are expanded and exhibit a distinctive metabolic state with enhanced glycolysis and OxPhos pathways as well as upregulated expression of granule proteases. We have also revealed that the CD11b^+^Ly6C^high^ monocyte population as a whole is more phagocytic in sepsis-surviving mice compared to sham-operated mice, and the classical monocyte subpopulation is especially involved in this enhancement of phagocytosis via increased FcγRI expression. Finally, this study suggests that CD11b^+^Ly6C^high^ M-MDSCs contribute to extramedullary myelopoiesis and immunosuppression due to metabolic reprogramming in sepsis-surviving mice.

## Supplementary Information

Supplementary Material 1. Figure 1. Gating strategy for scRNA-seq. Viable splenic monocytes purified by negative selection as described in Methods (routinely <2% Ly6G+) were sorted to obtain pure CD11b+Ly6Chigh cells for scRNA-seq analysis. Figure 2. Gating Strategy, masking, and phagocytosis of non-opsonized E.coli in phagocytosis assay. (A, B) Gating strategy for analysis of phagocytosis of (A) SRBCs and (B) E.coli particles. Among single focused CD11b+Ly6Chigh cells, SRBCs+ or E. coli+ cells were gated using Fluorescence Minus One Controls (FMO). (C) Then internalized SRBCs+ or E. coli+ were evaluated by using a ‘morphology’ mask for CD11b. (D, E) Mean fluorescent intensity of internalized non-opsonized E.coli particles in (D) C57BL/6J (n=4, Sham; n=5 CLP) and (E) BALB/c (n= 4, Sham; n=7 CLP) mice, respectively. Data are presented as mean ± SEM. Sham vs. CLP NS, not significant (Mann-Whitney test). Figure 3. CD11b+Ly6Chigh exhibits enhanced glycolysis in CLP surviving BALB/c mice. The extracellular acidification rate (ECAR) and oxygen consumption rate (OCR) in sort purified CD11b+Ly6Chigh cells from sham and CLP surviving BALB/c mice at 4 weeks post-surgery were assessed by the Seahorse Glycolysis Stress assay following glucose, oligomycin and 2-DG treatments. GLUT1 expression and 2-NBDG uptake were determined by flow cytometry. Arg1 expression was determined by qPCR. (A) Baseline ECAR and relative change in ECAR in CD11b+Ly6Chigh cells from sham and CLP surviving BALB/c mice. (B) Average rate of glycolysis in CD11b+Ly6Chigh cells from sham and CLP surviving BALB/c mice (± SD; n=3 Sham; n=3 CLP). (C) OCR-ECAR energy maps at baseline and after addition of glucose. (D) Percent expression of GLUT1 on BALB/c CD11b+Ly6Chigh cells (±SEM; n=14, Sham; n=15 CLP). (E) Glucose uptake in CD11b+Ly6Chigh cells from sham and CLP surviving BALB/c mice (± SD; n=3 Sham; n=5 CLP). (F) Quantitative PCR analysis of Arg1 in CD11b+Ly6Chigh cells from sham and CLP surviving BALB/c mice (± SD; n=4 Sham; n=5 CLP). Data plotted in panels A-C are from one of 3 representative experiments. Sham vs. CLP *P < 0.05,***P < 0.001, ****P < 0.0001 (unpaired t-test for panel B, Mann-Whitney test for panels D,E, unpaired t-test of log transformed data for panel F). Figure 4. Genes used for cluster annotation. The dot plot was visualized using CD11b+Ly6Chigh cells from sham and sepsis-surviving (CLP) mice of both C56BL/6J and BALB/c strains. The percentage of gene expression in all the cells of a specific cluster is represented by the size of the dot. The color reflects the level of expression of the gene. Figure 5. M-MDSC like CD11b+Ly6Chigh cells exhibit neutrophil-like features. The dot plot was visualized using CD11b+Ly6Chigh cells from sham and sepsis-surviving (cecal ligation and puncture) mice of both C56BL/6J and BALB/c strains. The percentage of gene expression in all the cells of a specific cluster is represented by the size of the dot. The color reflects the level of expression of the gene (Elane, Prtn3, Ctsg, Mpo, Lcn2, Fcnb, Camp). Figure 6. UCell score analysis for glycolysis in expanded subpopulations of CD11b+Ly6Chigh cells in BALB/c sepsis-surviving mice. (A) Violin plots and (B) bar graphs showing UCell score of glycolysis in sham and CLP in BALB/c mice. UCell scores for glycolysis were calculated using genes from the REATOME pathway (R-MMU-70171 and Table S8). (C) Cluster 8 in CLP show higher levels than other clusters within CLP and all clusters in sham. Sham vs. CLP ****P < 0.0001; NS, not significant (Mann-Whitney test). Figure 7. UCell score analysis for oxidative phosphorylation in expanded subpopulations of CD11b+Ly6Chigh cells in C57BL/6J and BALB/c sepsis- surviving mice. (A) Violin plots and (B) bar graphs showing UCell score of oxidative phosphorylation (OxPhos) in sham and CLP in C57BL/6J mice. UCell scores for OxPhos were calculated using genes from the KEGG pathway (mmu00190 and Table S6). (C) Clusters 8,11 and 12 in CLP show higher levels than other clusters within CLP and all clusters in C57BL/6J sham mice. (D) Violin plots and (E) bar graphs showing UCell score of OxPhos in sham and CLP in BALB/c mice. UCell scores for OxPhos were calculated using genes from gs_OxPhos (GO and Table S8). (F) Clusters 8,11 and 12 in CLP show higher levels than other clusters within CLP and all clusters in BALB/c sham mice. Sham vs. CLP **P < 0.01; ***P < 0.001; ****P < 0.0001 (Mann-Whitney test). Figure 8. Protease genes are upregulated in clusters 8, 11 and 12 in C57BL/6J sepsis-surviving mice. In clusters 8, 11, and 12 higher levels of Elane, Prtn3, Lcn2 and Fcnb are observed in CLP mice compared to sham-operated mice. *P < 0.05; ***P < 0.001; ****P < 0.0001 (Mann-Whitney test). Figure 9. Protease genes are upregulated in clusters 8, 11 and 12 in BALB/c sepsis-surviving mice. In clusters 8, 11, and 12 higher levels of Elane, Prtn3, Lcn2 and Fcnb are observed in CLP mice compared to sham-operated mice. *P < 0.05; ***P < 0.001; ****P < 0.0001 (Mann-Whitney test).

Supplementary Material 2. Table 1. Summary of quality control. (*.xlsx). Table 2. Frequency of cell sets. (*.xlsx). Table 3. Cell Set Frequency Statistics (13 clusters). (*.xlsx). Table 4. Marker genes for each cluster of C57BL/6J mice. (*.xlsx). Table 5. Combined Cell Set Frequency Statistics (7 subsets). (*.xlsx). Table 6. List of differentially expressed genes between sham vs. CLP in each cluster of C57BL/6J mice. (*.xlsx). Table 7. Lists of glycolysis and oxidative phosphorylation genes for UCell scores. (*.xlsx). Table 8. List of differentially expressed genes between sham vs. CLP in each cluster of BALB/c mice. (*.xlsx)

## Data Availability

The data that support the findings of this study are available in the GEO database at (https://www.ncbi.nlm.nih.gov/geo/query/acc.cgi?acc=GSE249839), reference number (GSE249839). Further inquiries can be directed to the corresponding authors.

## Abbreviations

ADAM17: A disintegrin and metalloproteinase 17
APC: Allophycocyanin
CARS: Compensatory anti-inflammatory response syndrome
CCR2: CC chemokine receptor 2
CLP: Cecal ligation and puncture
CR: Complement receptor
CX3CR1: C-X3-C motif chemokine receptor 1
DEG: Differentially expressed genes
ECAR: Extracellular Acidification Rate
FBS: Fetal bovine serum
FcγR: Fc-gamma receptor
FITC: Fluorescein isothiocyanate
GLUT1: Glucose transporter 1
GMP: Granulocyte macrophage progenitors
GOBP: Gene Ontology Biological Process
IMC: Immature myeloid cell
LAIR-1: Leukocyte-associated immunoglobulin-like receptor 1
M-MDSC: Monocytic myeloid-derived suppressor cell
MDSC: Myeloid-derived suppressor cell
MERTK: Mer tyrosine kinase
MoDC: Monocyte-derived dendritic cell
OxPhos: Oxidative phosphorylation
P-MDSC: Polymorphonuclear myeloid-derived suppressor cell
PE: Phycoerythrin
s.c.: Subcutaneous injection
scRNA-seq: Single-cell RNA-seq
SRBC: Sheep red blood cells
UMAP: Uniform Manifold Approximation and Projection
