# Supplementary material for: Single cell RNA-seq reveals cellular and transcriptional heterogeneity in the splenic CD11b+Ly6Chigh monocyte population expanded in sepsis-surviving mice
Source: Mol Med. 2024 Nov 6;30:202. doi: 10.1186/s10020-024-00970-0 (PMC11539566; doi:10.1186/s10020-024-00970-0)
Supplement: Supplementary file 1 — Supplementary Material 1. Figure 1. Gating strategy for scRNA-seq. Viable splenic monocytes purified by negative selection as described in Methods (routinely <2% Ly6G+) were sorted to obtain pure CD11b+Ly6Chigh cells for scRNA-seq analysis. Figure 2. Gating Strategy, masking, and phagocytosis of non-opsonized E.coli in phagocytosis assay. (A, B) Gating strategy for analysis of phagocytosis of (A) SRBCs and (B) E.coli particles. Among single focused CD11b+Ly6Chigh cells, SRBCs+ or E. coli+ cells were gated using Fluorescence Minus One Controls (FMO). (C) Then internalized SRBCs+ or E. coli+ were evaluated by using a ‘morphology’ mask for CD11b. (D, E) Mean fluorescent intensity of internalized non-opsonized E.coli particles in (D) C57BL/6J (n=4, Sham; n=5 CLP) and (E) BALB/c (n= 4, Sham; n=7 CLP) mice, respectively. Data are presented as mean ± SEM. Sham vs. CLP NS, not significant (Mann-Whitney test). Figure 3. CD11b+Ly6Chigh exhibits enhanced glycolysis in CLP surviving BALB/c mice. The extracellular acidification rate (ECAR) and oxygen consumption rate (OCR) in sort purified CD11b+Ly6Chigh cells from sham and CLP surviving BALB/c mice at 4 weeks post-surgery were assessed by the Seahorse Glycolysis Stress assay following glucose, oligomycin and 2-DG treatments. GLUT1 expression and 2-NBDG uptake were determined by flow cytometry. Arg1 expression was determined by qPCR. (A) Baseline ECAR and relative change in ECAR in CD11b+Ly6Chigh cells from sham and CLP surviving BALB/c mice. (B) Average rate of glycolysis in CD11b+Ly6Chigh cells from sham and CLP surviving BALB/c mice (± SD; n=3 Sham; n=3 CLP). (C) OCR-ECAR energy maps at baseline and after addition of glucose. (D) Percent expression of GLUT1 on BALB/c CD11b+Ly6Chigh cells (±SEM; n=14, Sham; n=15 CLP). (E) Glucose uptake in CD11b+Ly6Chigh cells from sham and CLP surviving BALB/c mice (± SD; n=3 Sham; n=5 CLP). (F) Quantitative PCR analysis of Arg1 in CD11b+Ly6Chigh cells from sham and CLP surviving BALB/c mice [file 10020_2024_970_MOESM1_ESM.pptx]

## Slide 1
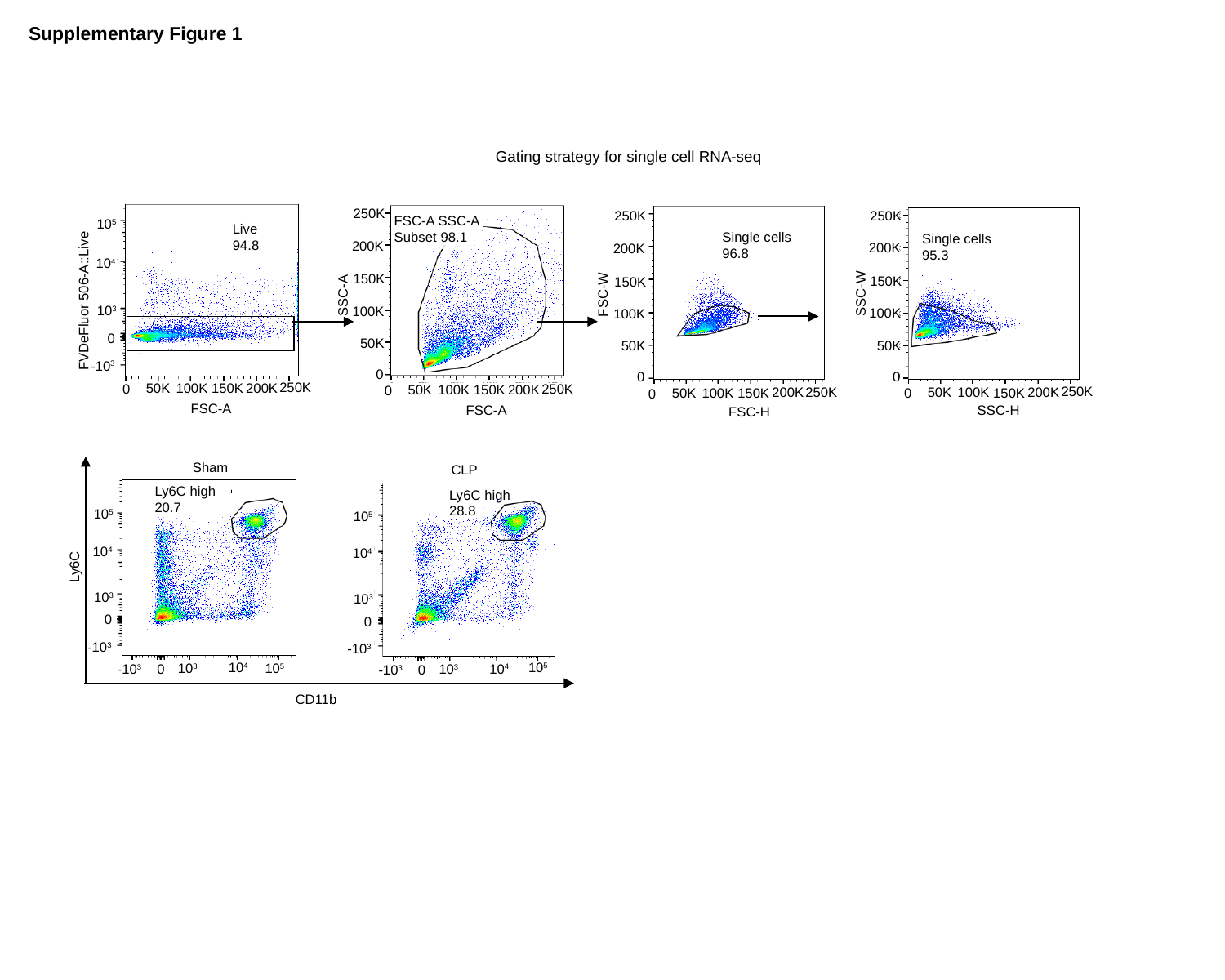

Supplementary Figure 1
Gating strategy for single cell RNA-seq
250K
200K
150K
100K
50K
0
250K
200K
150K
100K
50K
0
250K
200K
150K
100K
50K
0
105
104
103
0
-103
FSC-A SSC-A
Subset 98.1
Live
94.8
Single cells
96.8
Single cells
95.3
FVDeFluor 506-A::Live
SSC-A
SSC-W
FSC-W
250K
200K
50K
100K
150K
0
250K
200K
50K
100K
150K
0
250K
200K
50K
100K
150K
0
250K
200K
50K
100K
150K
0
FSC-A
SSC-H
FSC-A
FSC-H
Sham
CLP
Ly6C high
28.8
Ly6C high
20.7
105
104
103
-103
0
105
104
103
0
-103
105
104
103
0
-103
Ly6C
104
103
-103
0
105
CD11b

## Slide 2
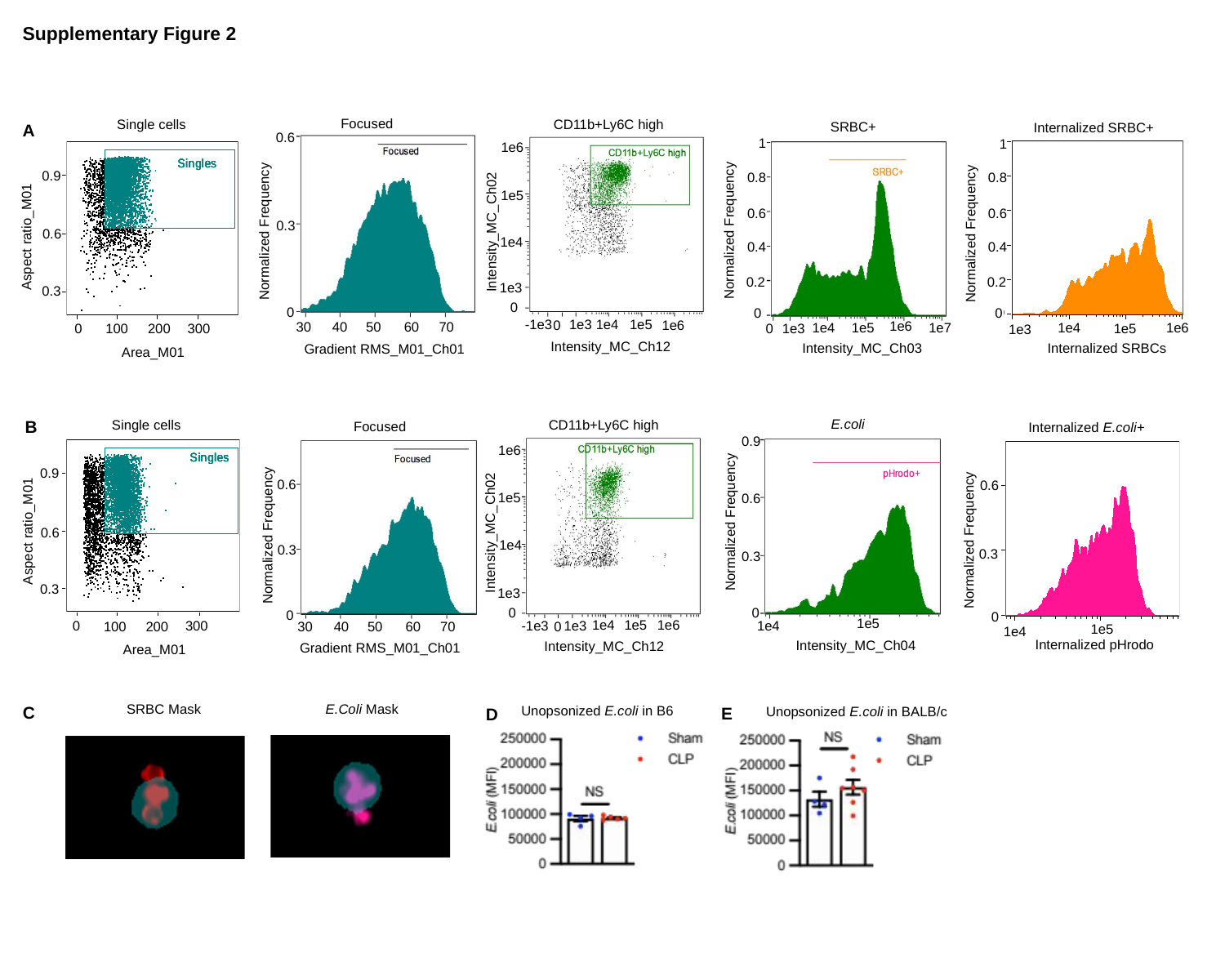

Supplementary Figure 2
Focused
CD11b+Ly6C high
Single cells
SRBC+
Internalized SRBC+
A
0.6
0.3
0
1
1
1e6
1e5
1e4
1e3
0
0.9
0.6
0.3
0
300
100
200
0.8
0.8
Aspect ratio_M01
Intensity_MC_Ch02
Normalized Frequency
Normalized Frequency
Normalized Frequency
0.6
0.6
0.4
0.4
0.2
0.2
0
0
-1e3
 1e5
 1e3
 1e4
 1e6
0
 1e6
40
50
60
70
30
 1e5
 1e4
 1e7
 1e4
 1e6
 1e3
 1e5
0
 1e3
Intensity_MC_Ch12
Internalized SRBCs
Intensity_MC_Ch03
Gradient RMS_M01_Ch01
Area_M01
E.coli
B
Single cells
CD11b+Ly6C high
Focused
Internalized E.coli+
0.9
1e6
1e5
1e4
1e3
0
0.9
0.6
0.3
0
300
100
200
0.6
0.3
0
0.6
Aspect ratio_M01
Normalized Frequency
Intensity_MC_Ch02
0.6
Normalized Frequency
Normalized Frequency
0.3
0.3
0
0
 1e5
 1e6
 1e5
 1e4
 1e3
 1e4
-1e3
 0
40
50
60
70
30
 1e5
 1e4
Internalized pHrodo
Intensity_MC_Ch04
Intensity_MC_Ch12
Gradient RMS_M01_Ch01
Area_M01
SRBC Mask
E.Coli Mask
Unopsonized E.coli in B6
C
E
Unopsonized E.coli in BALB/c
D

## Slide 3
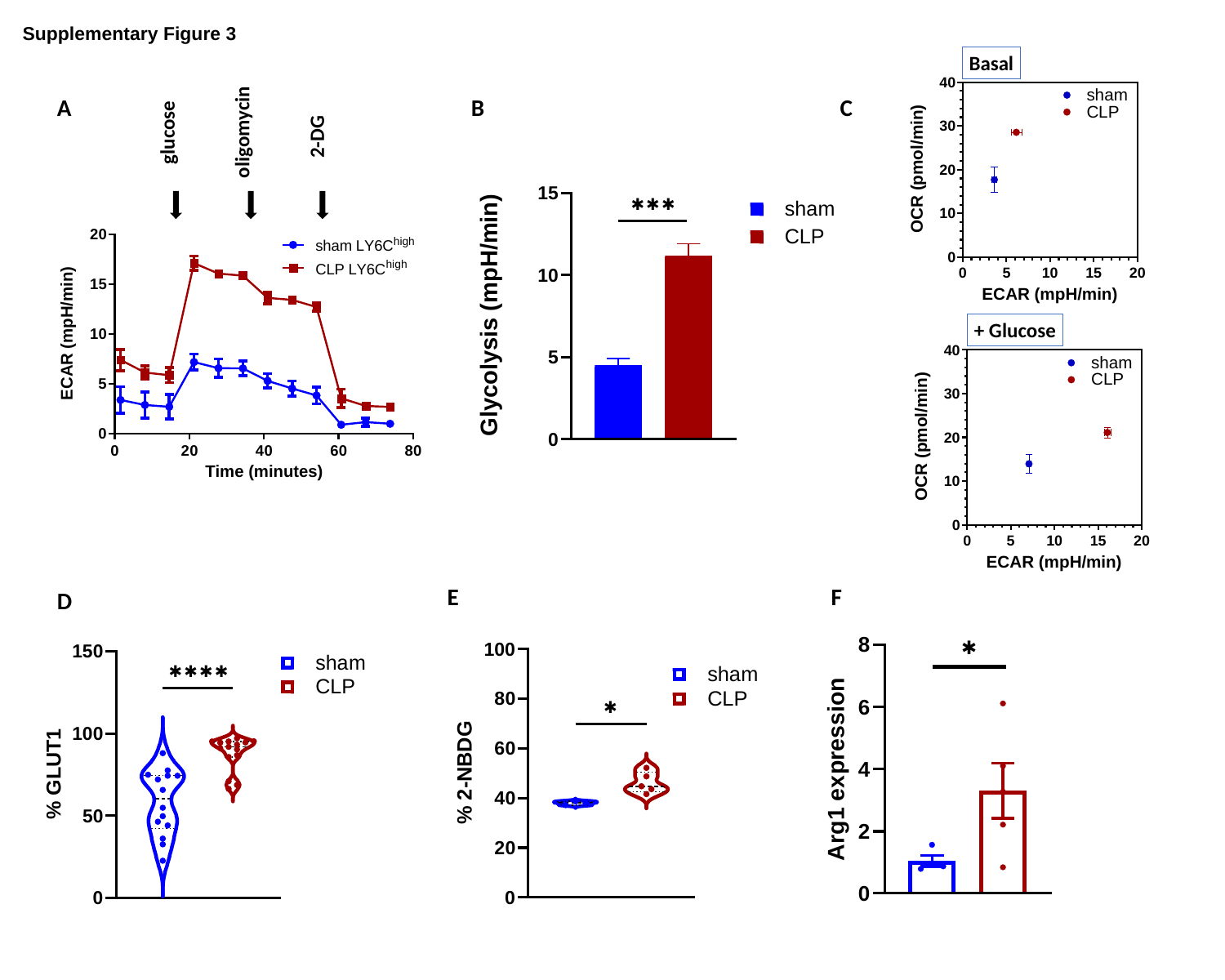

Supplementary Figure 3
Basal
oligomycin
A
B
C
glucose
2-DG
+ Glucose
E
F
D

## Slide 4
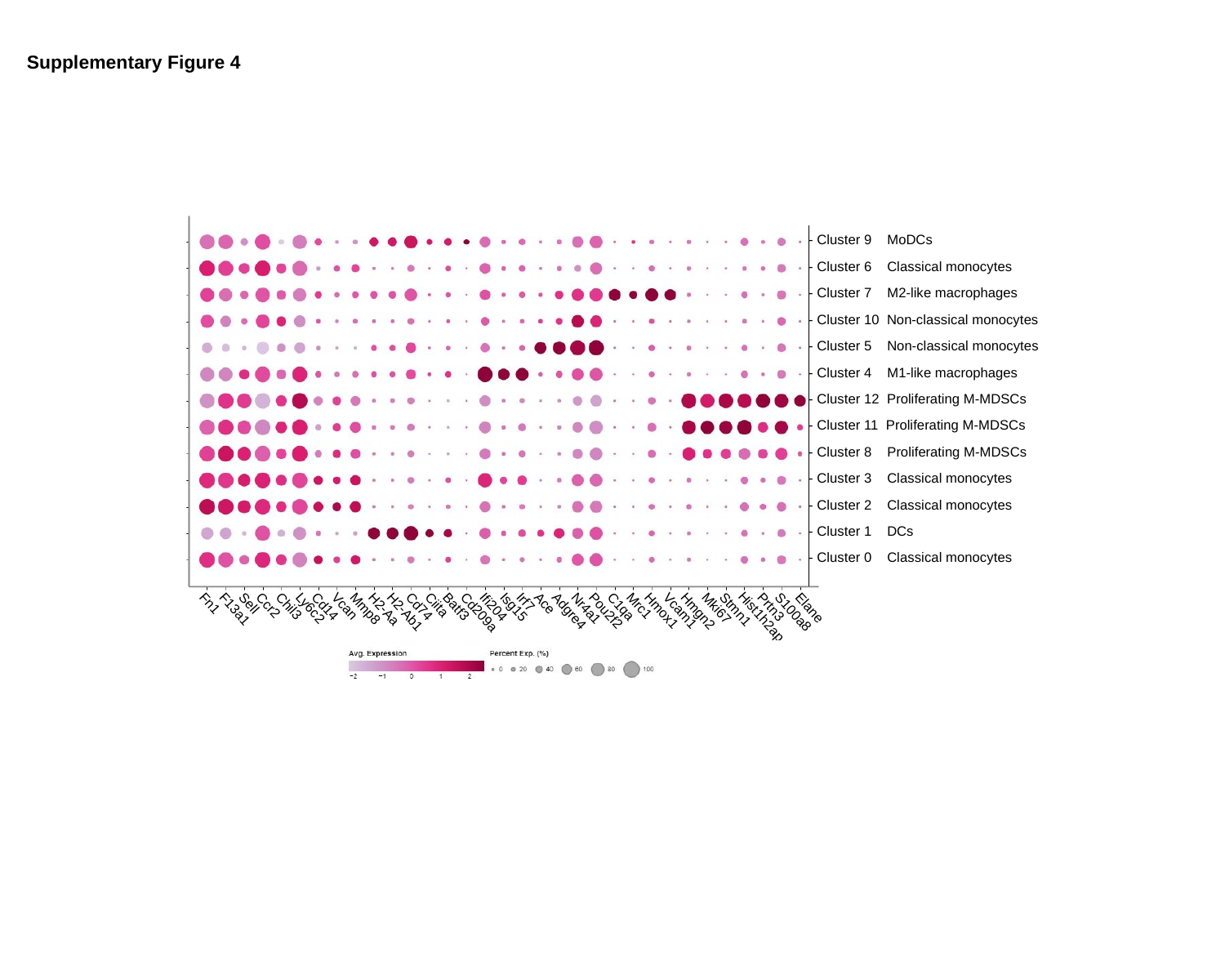

Supplementary Figure 4
- Cluster 9 MoDCs
- Cluster 6 Classical monocytes
- Cluster 7 M2-like macrophages
- Cluster 10 Non-classical monocytes
- Cluster 5 Non-classical monocytes
- Cluster 4 M1-like macrophages
- Cluster 12 Proliferating M-MDSCs
- Cluster 11 Proliferating M-MDSCs
- Cluster 8 Proliferating M-MDSCs
- Cluster 3 Classical monocytes
- Cluster 2 Classical monocytes
- Cluster 1 DCs
- Cluster 0 Classical monocytes
Fn1
F13a1
Sell
Ccr2
Chil3
Ly6c2
Cd14
Vcan
Mmp8
H2-Aa
Cd74
Ciita
Batf3
Ifi204
Isg15
Irf7
Ace
Nr4a1
C1qa
Mrc1
Hmox1
Vcam1
Mki67
Stmn1
Prtn3
Elane
S100a8
Cd209a
Adgre4
Hmgn2
H2-Ab1
Pou2f2
Hist1h2ap

## Slide 5
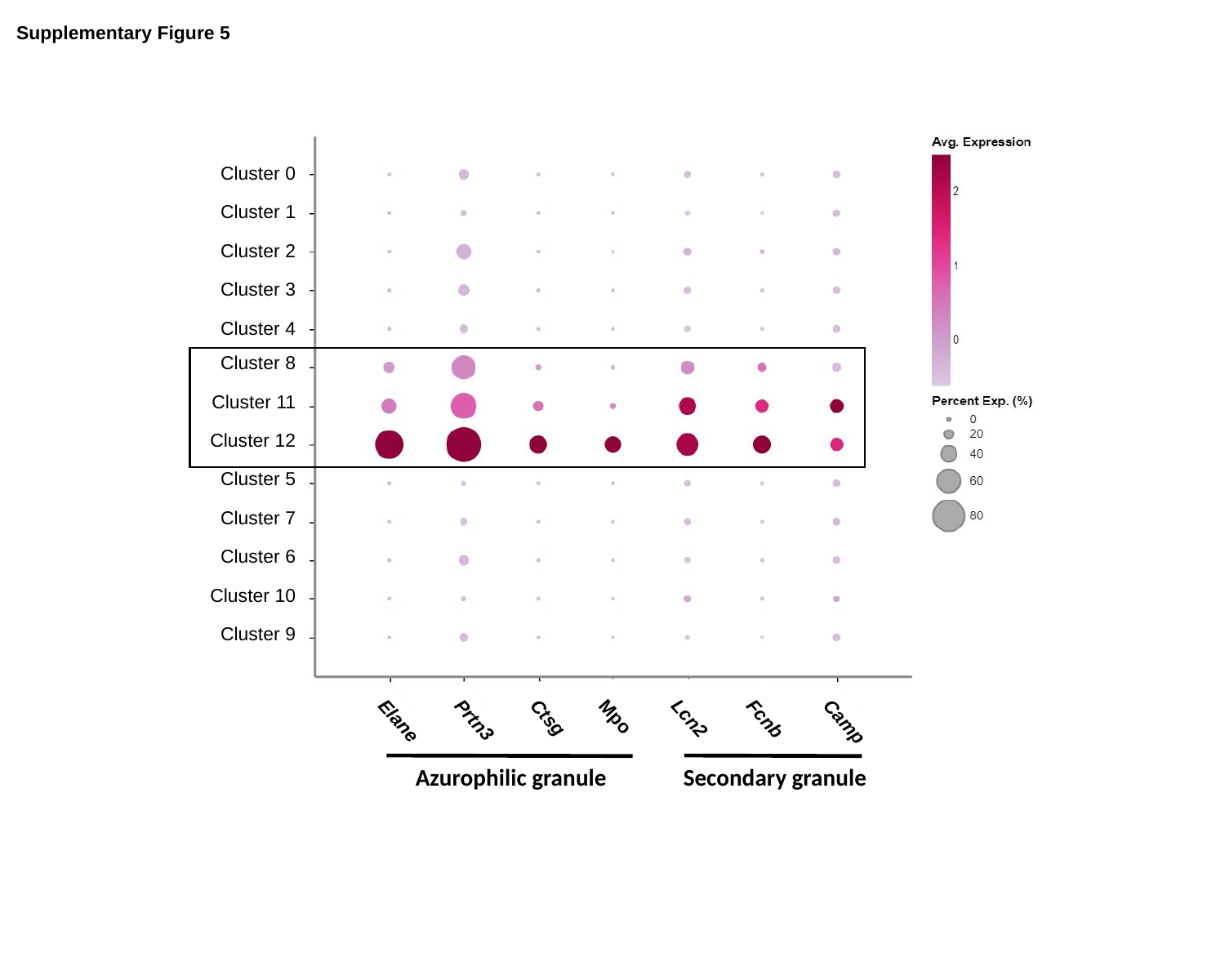

Supplementary Figure 5
Cluster 0
Cluster 1
Cluster 2
Cluster 3
Cluster 4
Cluster 8
Cluster 11
Cluster 12
Cluster 5
Cluster 7
Cluster 6
Cluster 10
Cluster 9
Lcn2
Fcnb
Camp
Mpo
Elane
Prtn3
Ctsg
Mpo
Lcn2
Fcnb
Camp
Secondary granule
Azurophilic granule

## Slide 6
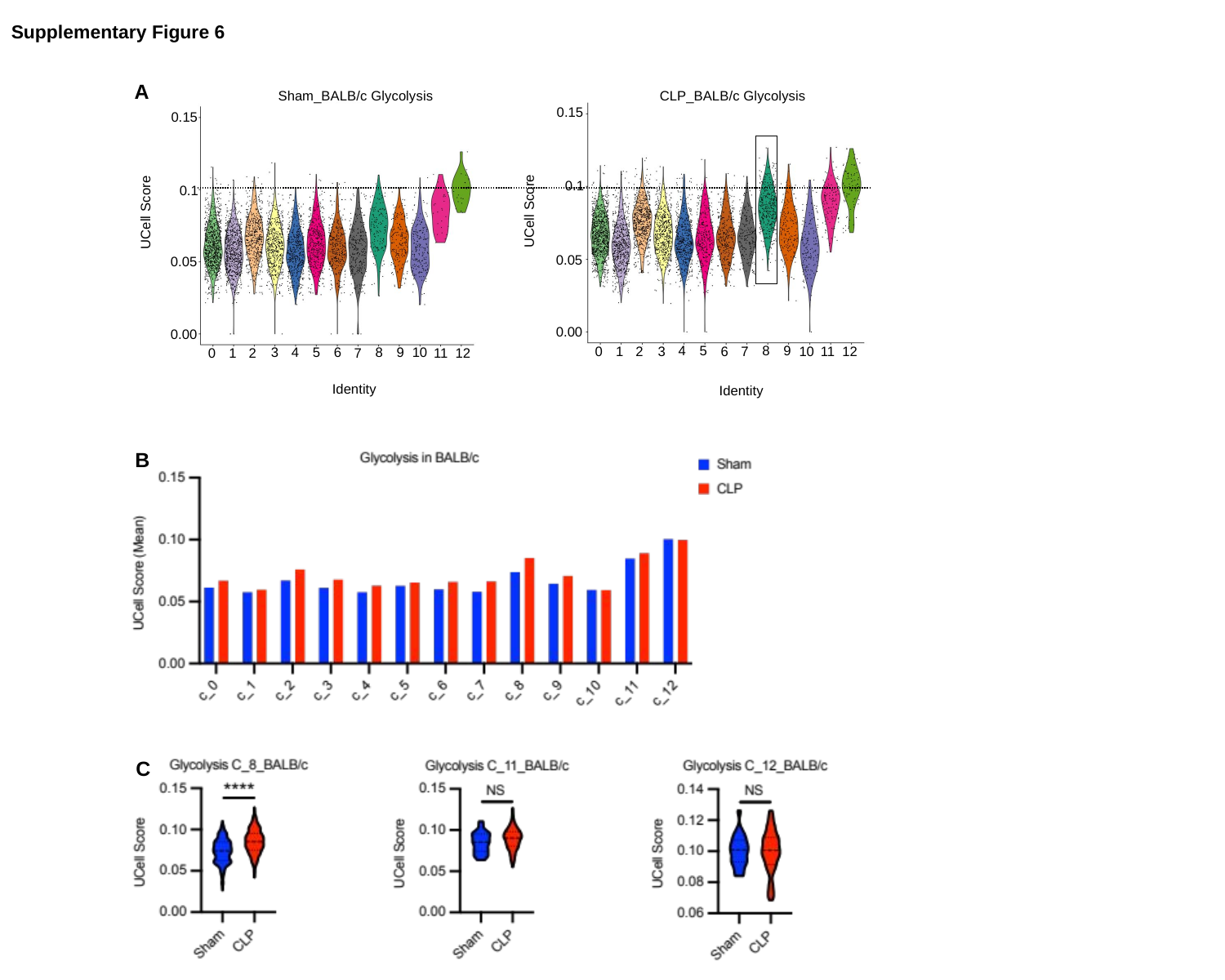

Supplementary Figure 6
A
Sham_BALB/c Glycolysis
CLP_BALB/c Glycolysis
0.15
0.15
UCell Score
UCell Score
0.1
0.1
0.05
0.05
0.00
0.00
5
9
4
8
3
6
10
7
11
12
0
2
1
5
9
4
8
3
6
10
7
11
12
0
2
1
Identity
Identity
B
C

## Slide 7
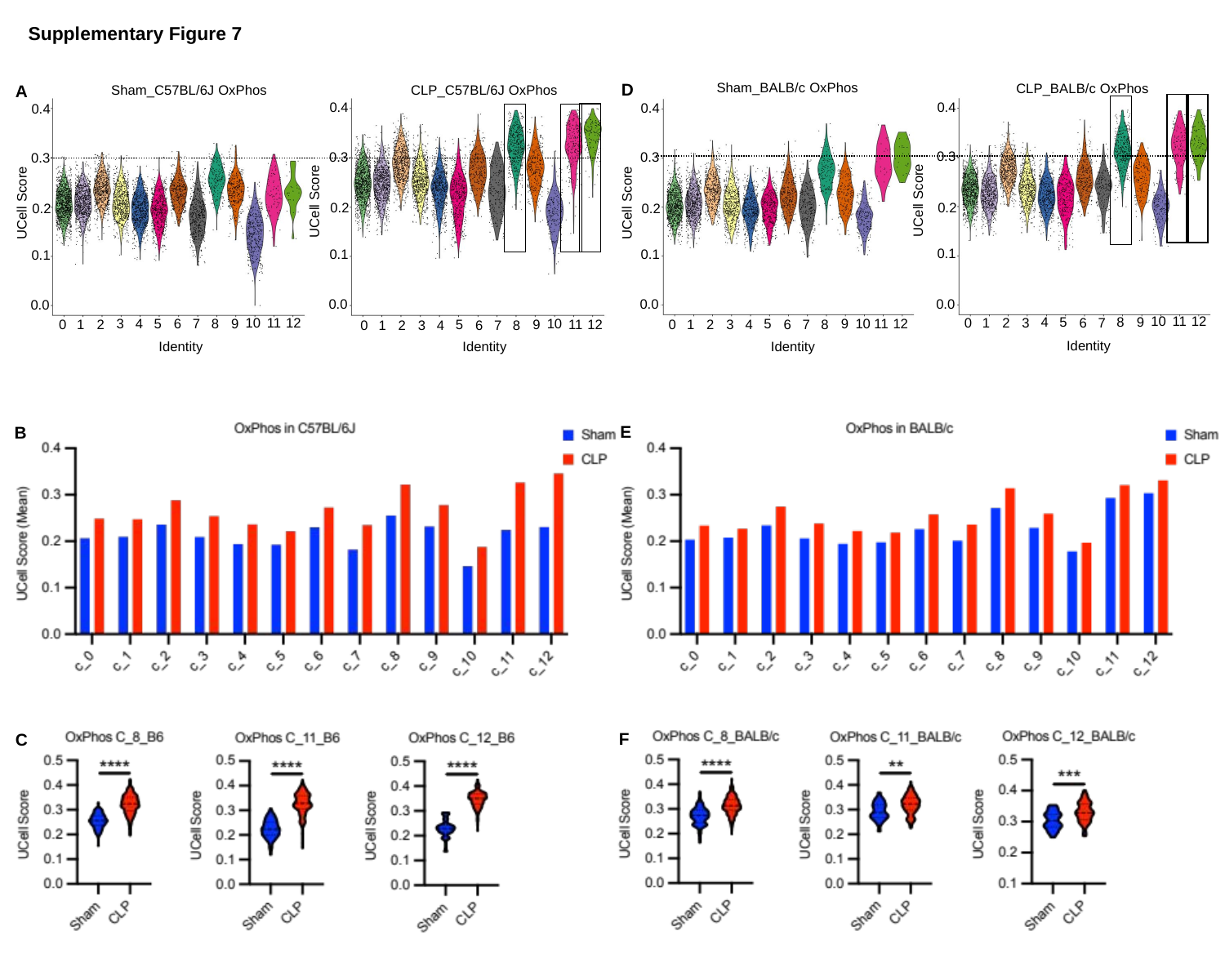

Supplementary Figure 7
Sham_BALB/c OxPhos
D
CLP_BALB/c OxPhos
A
Sham_C57BL/6J OxPhos
CLP_C57BL/6J OxPhos
0.4
0.3
0.2
0.1
0.0
0.4
0.3
0.2
0.1
0.0
0.4
0.3
0.2
0.1
0.0
0.4
0.3
0.2
0.1
0.0
UCell Score
UCell Score
UCell Score
UCell Score
10
11
12
5
9
4
8
3
6
7
10
11
12
0
2
1
10
5
9
11
12
4
8
3
6
7
5
9
10
11
12
0
2
1
4
8
3
6
7
5
9
0
2
1
4
8
3
6
7
0
2
1
Identity
Identity
Identity
Identity
E
B
F
C

## Slide 8
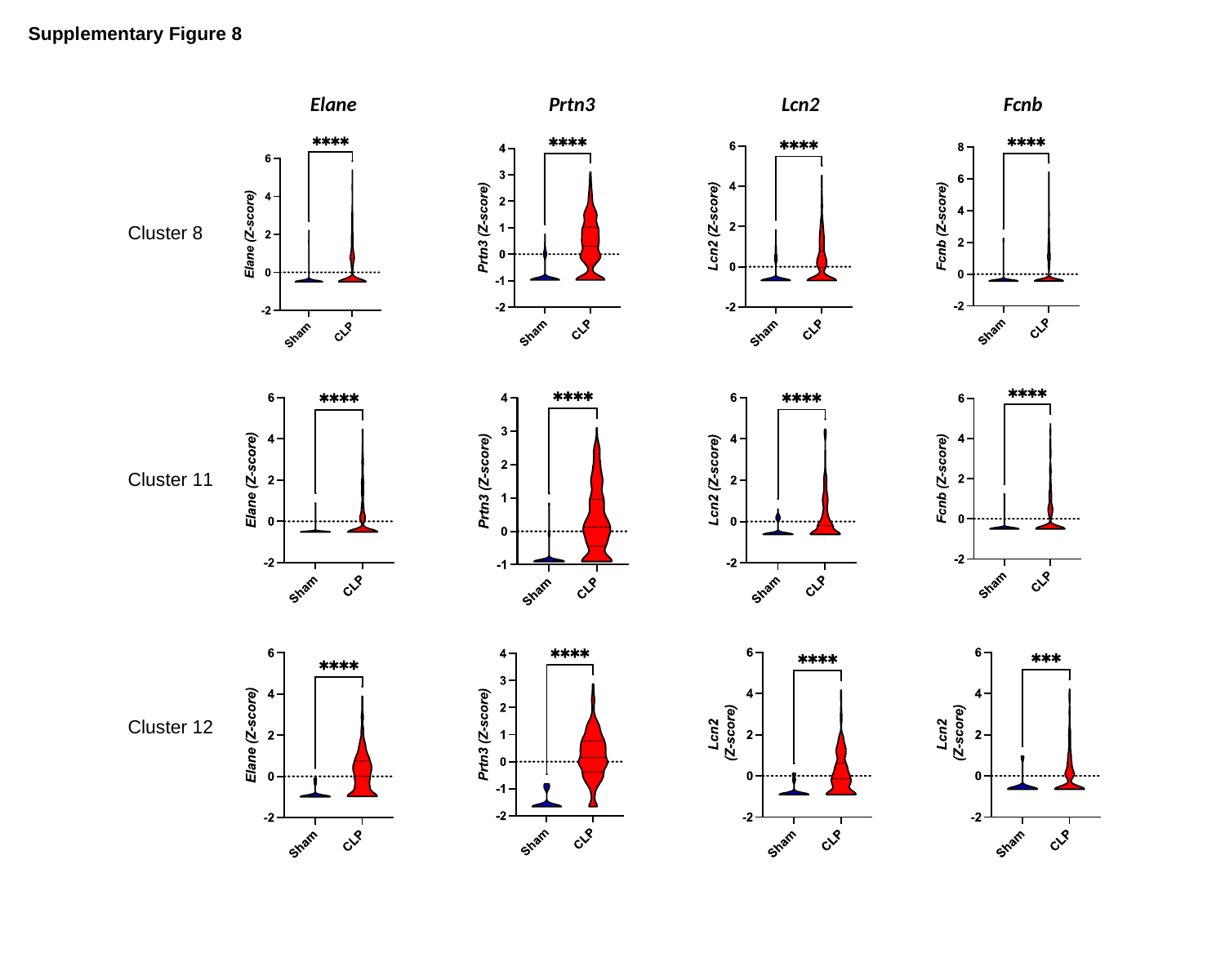

Supplementary Figure 8

## Slide 9
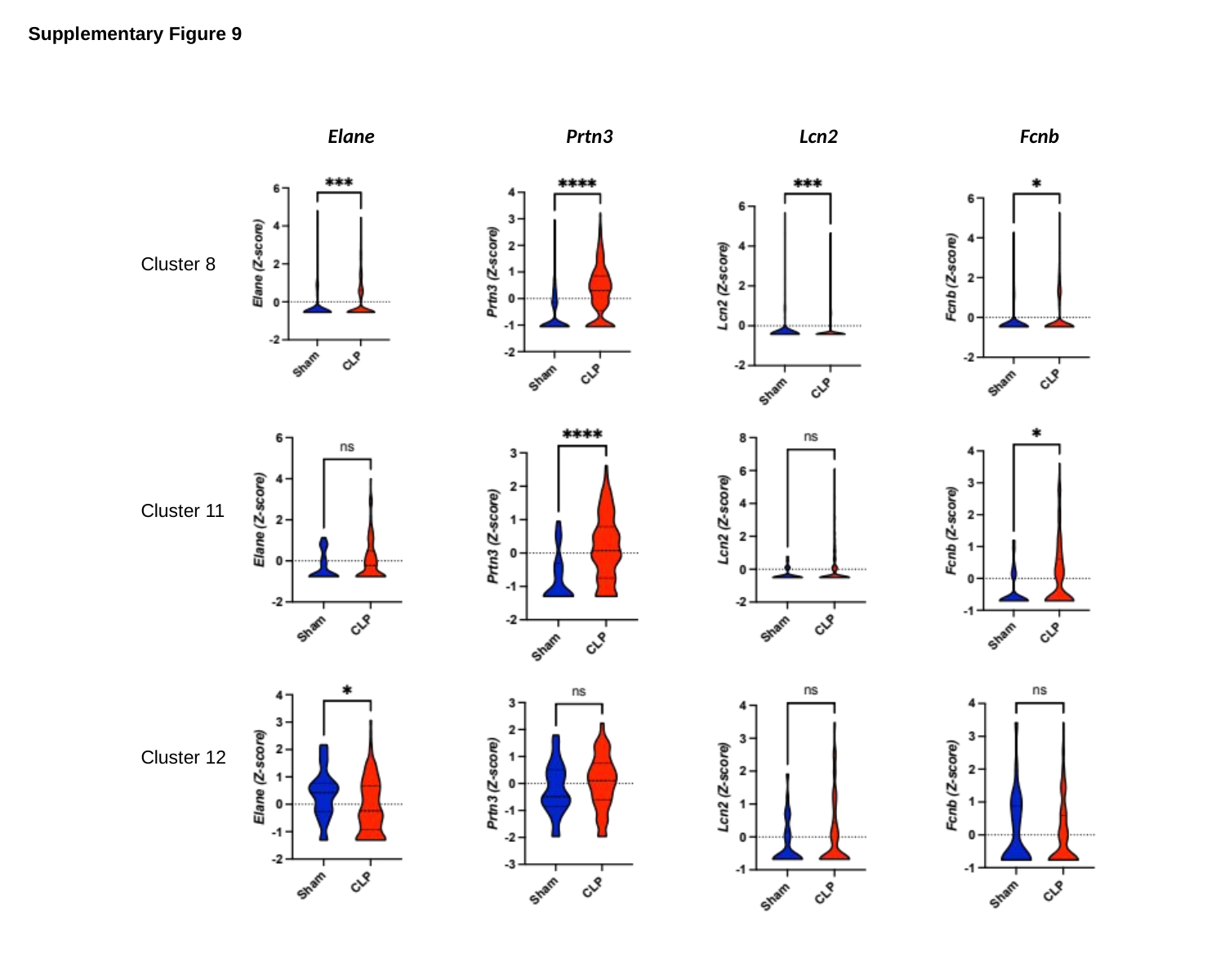

Supplementary Figure 9
Elane Prtn3 Lcn2 Fcnb
Cluster 8
Cluster 11
Cluster 12
